# Tinnitus in urban settings: insights into lifestyle and medical factors from the Hamburg City Health Study

**DOI:** 10.1007/s00405-026-10518-z

**Published:** 2026-09-29

**Authors:** Benjamin Becker, Konrad Reuter, Mark Praetorius, Ines Schäfer, Hanno Hoven, Volker Harth, Birgit-Christiane Zyriax, Petra Brüggemann, Birgit Mazurek, Martin Härter, Amra Pepić, Christian Betz, Anna Sophie Hoffmann

**Affiliations:** 1 Department of Otorhinolaryngology, Head and Neck Surgery and Oncology, University Medical Center Hamburg-Eppendorf, Hamburg, Germany; 2 Population Health Research Department, University Heart and Vascular Center, University Medical Center Hamburg-Eppendorf, Hamburg, Germany; 3 Institute for Occupational and Maritime Medicine (ZfAM), University Medical Center Hamburg- Eppendorf, Hamburg, Germany; 4 Research Group Preventive Medicine and Nutrition, Midwifery Science-Health Care Research and Prevention, University Medical Center Hamburg-Eppendorf, Hamburg, Germany; 5 Tinnitus Center, Charité, University Medical Center Berlin, Berlin, Germany; 6 Institute of Medical Technology and Intelligent Systems, Hamburg University of Technology, Hamburg, Germany; 7 Department of Medical Psychology, University Medical Center Hamburg-Eppendorf, Hamburg, Germany; 8 Institute of Medical Biometry and Epidemiology, University Medical Center Hamburg-Eppendorf, Hamburg, Germany; 9 Department of Otorhinolaryngology, Head and Neck Surgery and Oncology, University Medical Center Hamburg-Eppendorf, Martinistraße 52, 20251 Hamburg, Germany

**Keywords:** Tinnitus / epidemiology, Hearing Loss, Risk Factors, Environmental Noise, Urban Population, Cross-Sectional Studies

## Abstract

**Purpose:**

Tinnitus is a common and multifactorial auditory condition associated with considerable psychosocial burden. Evidence regarding its relationship with demographic, lifestyle, and medical factors remains inconsistent. This study aimed to determine the prevalence and correlates of tinnitus in an urban Western European population, focusing on lifestyle, socioeconomic, and clinical characteristics within the Hamburg City Health Study (HCHS).

**Methods:**

We conducted a population-based cross-sectional analysis of data from 14,355 participants aged 45-74 years enrolled in the HCHS. Tinnitus status was defined by self-reported medical diagnosis. For statistical analyses we fitted age- and sex-adjusted logistic regression models with tinnitus status as the binary outcome. Odds ratios (ORs) and corresponding 95% confidence intervals (95% CI) were derived from the model coefficients. Associations were examined across domains including demographics, comorbidities, medication, subjective hearing difficulties, and environmental noise exposure.

**Results:**

The lifetime self-reported physician-diagnosed tinnitus was 19.3%. Male sex, older age, previous ENT surgery, sensorineural and conductive hearing loss, antidepressant use, night-shift work, sleep apnea, carotid stenosis, migraines, and shingles were associated with tinnitus. Participants with tinnitus reported greater hearing difficulties, more pronounced social consequences of hearing problems, and higher perceived environmental noise exposure. No relevant associations were observed for income, education, alcohol consumption, or occupational noise exposure.

**Conclusion:**

Tinnitus in urban populations is linked to auditory and psychological comorbidities and perceived environmental noise, but not to traditional socioeconomic factors. These findings highlight the multifactorial nature of tinnitus and emphasize the need for integrated medical, audiological, and psychosocial management approaches.

**Supplementary Information:**

The online version contains supplementary material available at https://doi.org/10.1007/s00405-026-10518-z.

## Introduction

Tinnitus is characterized by the perception of sound without the presence of an external auditory stimulus. Individuals affected by tinnitus frequently report a range of associated symptoms, including frustration, irritability, anxiety, depression, hearing impairment, hyperacusis, insomnia, and difficulties with concentration. These comorbid symptoms play a critical role in assessing the severity of tinnitus [1–6]. Consequently, tinnitus is a highly prevalent and potentially debilitating condition with a wide range of symptoms that can place a huge burden on patients and substantially reduce quality of life. Its socioeconomic impact is underscored by the significantly increased risk of receiving a disability pension among individuals with tinnitus [7–9].

The prevalence of tinnitus and its associations with demographic, socioeconomic, and health-related factors – such as age, sex, marital status, employment status, socioeconomic status, educational attainment, lifestyle behaviors, and comorbid conditions – have not been comprehensively evaluated. A systematic review identified a total of 32 relevant studies on the topic [10]. Another systematic review and meta-analysis estimated a pooled global prevalence of any tinnitus among adults of 14.4% [11]. In the United States, estimates of tinnitus prevalence range from 8% to 25% of the general population [12]. A multinational European report in 12 countries showed a tinnitus prevalence of 14.7%, ranging from 8.7% in Ireland to 28.3% in Bulgaria [13, 14]. Several studies have reported a higher prevalence of tinnitus among males compared to females, both in the general population [13, 15, 16] and in individuals with noise-induced hearing loss [17, 18]. However, conflicting findings have also been reported; some studies suggest a higher likelihood of tinnitus among females [19], while others have found no significant association between sex and tinnitus prevalence [11, 20–22].

Several studies have identified associations between individuals’ educational attainment and employment status and the severity of tinnitus symptoms [23, 24]. Moreover, tinnitus has been shown to correlate with productivity losses that increase in proportion to the severity of the condition [25]. Research indicates that greater tinnitus-related disability is linked to higher healthcare and management costs [25]. Consequently, it has been suggested that individuals from lower socioeconomic backgrounds may experience disproportionately greater burdens due to tinnitus, including reduced access to effective treatment and greater losses in productivity and income [26].

A population-based cross-sectional analysis was conducted using data from the PERSIAN Guilan Cohort Study (PGCS) [27], which included 10,520 men and women aged 35 to 70 years from both urban and rural areas of “Sowme’e Sara” located in Guilan province in northern Iran. The study has shown that tinnitus was not associated with marital status, educational level, employment, socioeconomic status (SES), alcohol consumption, BMI, level of physical activity, sleep duration, hypertension, and diabetes. Moreover, adjusted analyses revealed no significant difference in tinnitus prevalence between male and female participants. However older age, residency in a rural area, cigarette smoking and having other comorbidities (odds ratio: 2.75, 95% confidence interval: 2.19–3.44) were related to tinnitus.

Serveral risk factors tinnitus have been identified in previous epidemiological studies [28, 29]. Prevalence increases significantly with advancing age, noise exposure and hearing loss. Specific risk factors include: sensorineural hearing loss, chronic noise exposure, stress and psychological strain, sleep disorders, temporomandibular joint and cervical spine dysfunctions, migraine and concomitant neurodegenerative conditions.

The literature also points to the increasing relevance of recreational noise and personal audio devices among younger populations. Another systematic review investigated the influence of lifestyle-related factors on tinnitus included 384 studies, the majority of which employed cross-sectional designs [30]. The findings indicated a significantly increased risk of tinnitus among current smokers (based on 26 studies), individuals with a history of smoking (based on 16 studies), and those who are obese (based on seven studies). However, no significant association was found between alcohol consumption (based on 11 studies). With respect to caffeine intake or coffee consumption, only three studies examined this factor, preventing any conclusive statements from being made. Recent literature indicates that tinnitus is influenced not only by auditory injury but also by modifiable lifestyle factors [31]. Preventive strategies should therefore emphasize hearing protection, healthy sleep habits, stress management, smoking cessation, regular physical activity, and reduction of harmful environmental exposures [32]. Such measures may decrease both the risk of tinnitus onset and the severity of tinnitus-related distress [33].

Despite extensive research on tinnitus, important uncertainties remain regarding its epidemiology and associated risk factors. Previous studies have reported inconsistent findings, particularly concerning the prevalence of tinnitus, its origin, long-term course, psychosocial dimensions and its associations with various related clinical and demographic factors. Notably, there is considerable divergence in the literature concerning the role of socioeconomic factors and gender. In light of these inconsistencies and the limited data available from large European population-based cohorts, the present study aims to contribute to the growing body of knowledge by examining these associations within a large cohort from Germany, specifically the HCHS cohort in a major German city. This will allow for a more comprehensive analysis of potential factors associated with tinnitus in a diverse high-participation sample. In an urban population, the overall prevalence of tinnitus is likely to be higher than the European average. Due in particular to higher noise exposure, but also to greater socio-economic stress and lifestyle factors, as well as other risks, we anticipate that the overall incidence of tinnitus severity in our cohort will be above average. Given the exploratory nature of this study and the inconsistent findings reported in previous literature, we aimed to investigate whether demographic, socioeconomic, lifestyle, medical, hearing-related, and environmental factors are associated with self-reported tinnitus in the Hamburg City Health Study cohort. Specifically, our study aimed to investigate the relationship between lifestyle factors, hearing problems and medical factors. Demographic and socioeconomic characteristics, including age, sex, marital status, educational level and employment status, are examined to determine whether specific demographic or socioeconomic trends are observed among individuals with tinnitus compared with the total study cohort. A better understanding of these associations may help identify populations at increased risk and contribute to the development of more targeted prevention and management strategies.

## Materials and methods

This study is a population-based cross-sectional analysis of baseline questionnaire data obtained from participants aged 45–74 years of the Hamburg City Health Study (HCHS), a large prospective cohort study conducted in Hamburg, Germany. The present investigation used retrospectively analyzed questionnaire data collected between 2017 and 2025 to examine associations between self-reported physician-diagnosed tinnitus and a broad range of demographic, socioeconomic, lifestyle, medical, hearing-related, and environmental factors.

A comprehensive description of the study design has been published elsewhere [34]. The first Data set consisting of 14,355 HCHS participants is analysed in this study. To differentiate between patients with and without tinnitus, the question: “Have you ever been diagnosed with tinnitus by a doctor?” was evaluated. Possible responses were “Yes”, “No”, “I don’t know” and “I don’t want to answer”. Only participants who responded “Yes” or “No” were included in the present analyses, leading to 2,765 participants with and 11,590 participants without tinnitus. The statistical analyses presented in this article were explorative and retrospective in nature and are based on data collected through structured questionnaires between 2017 and 2025. No adjustment for multiple testing was performed.

All Analyses of the HCHS cohort data were conducted using Python 3.12.7 with Pandas 2.2.2 and Numpy 1.26.4 for data management and statsmodels 0.14.2 for statistical testing. For each predictor, a separate age- and sex-adjusted logistic regression model was fitted, with tinnitus status as the binary outcome. Odds ratios (ORs) and corresponding 95% confidence intervals (95% CI) were derived from the model coefficients. Ordinal (Likert-scale) predictors were treated as continuous variables. Responses such as “I don’t know”, “I prefer not to answer”, and “I am not familiar with this situation” were treated as missing and excluded on a variable-specific basis. For categorical predictors, ORs indicate the odds of tinnitus relative to the reference category. For continuous predictors, ORs represent the multiplicative change in the odds of tinnitus per one-unit increase in the predictor. As each predictor was analyzed in a separate logistic regression model, the reported odds ratios represent age- and sex-adjusted associations for individual variables rather than mutually adjusted independent effects.

**Fig. 1:**
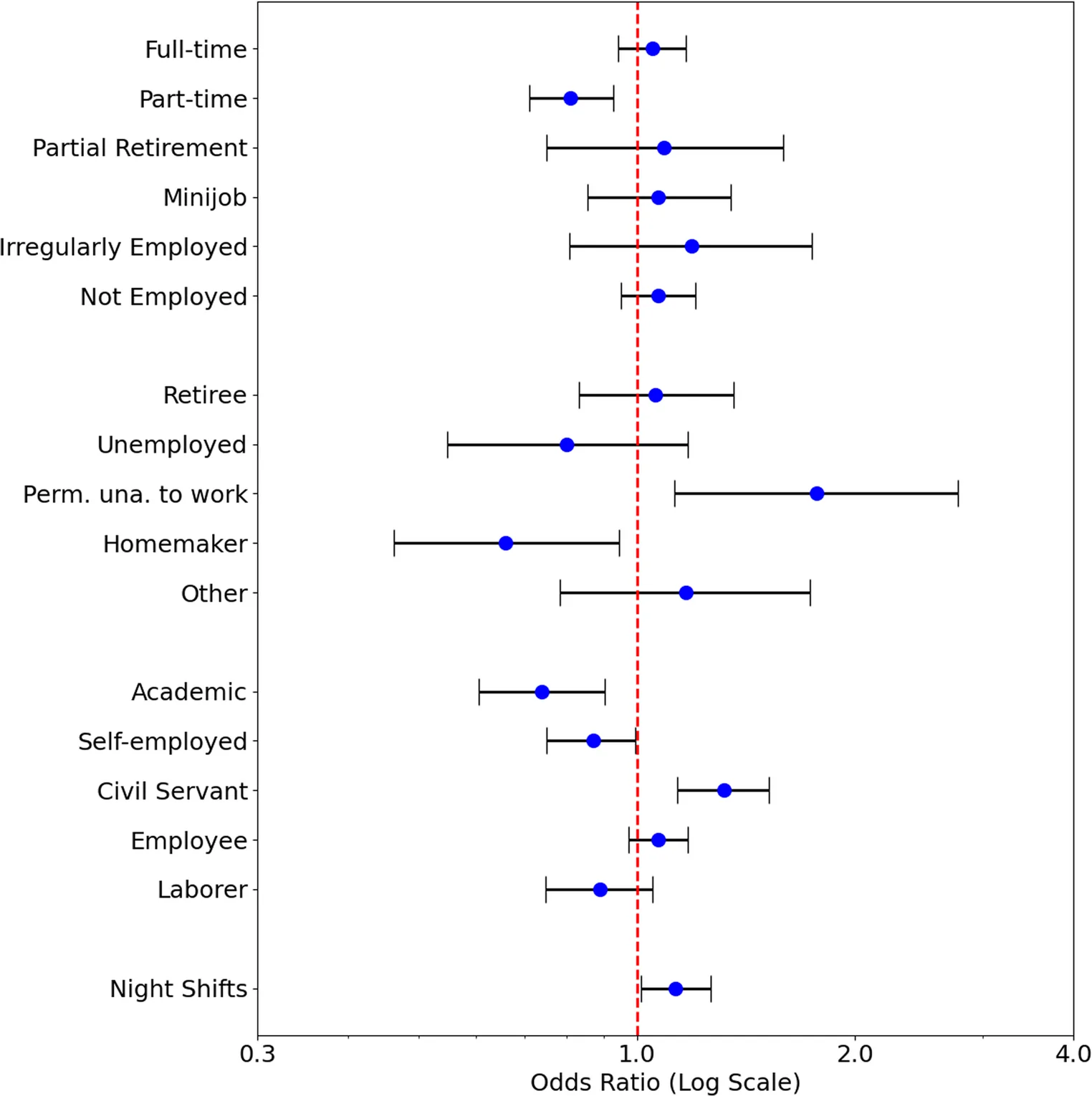
Odds ratios with 95% confidence intervals for demographic and lifestyle factors

**Fig. 2:**
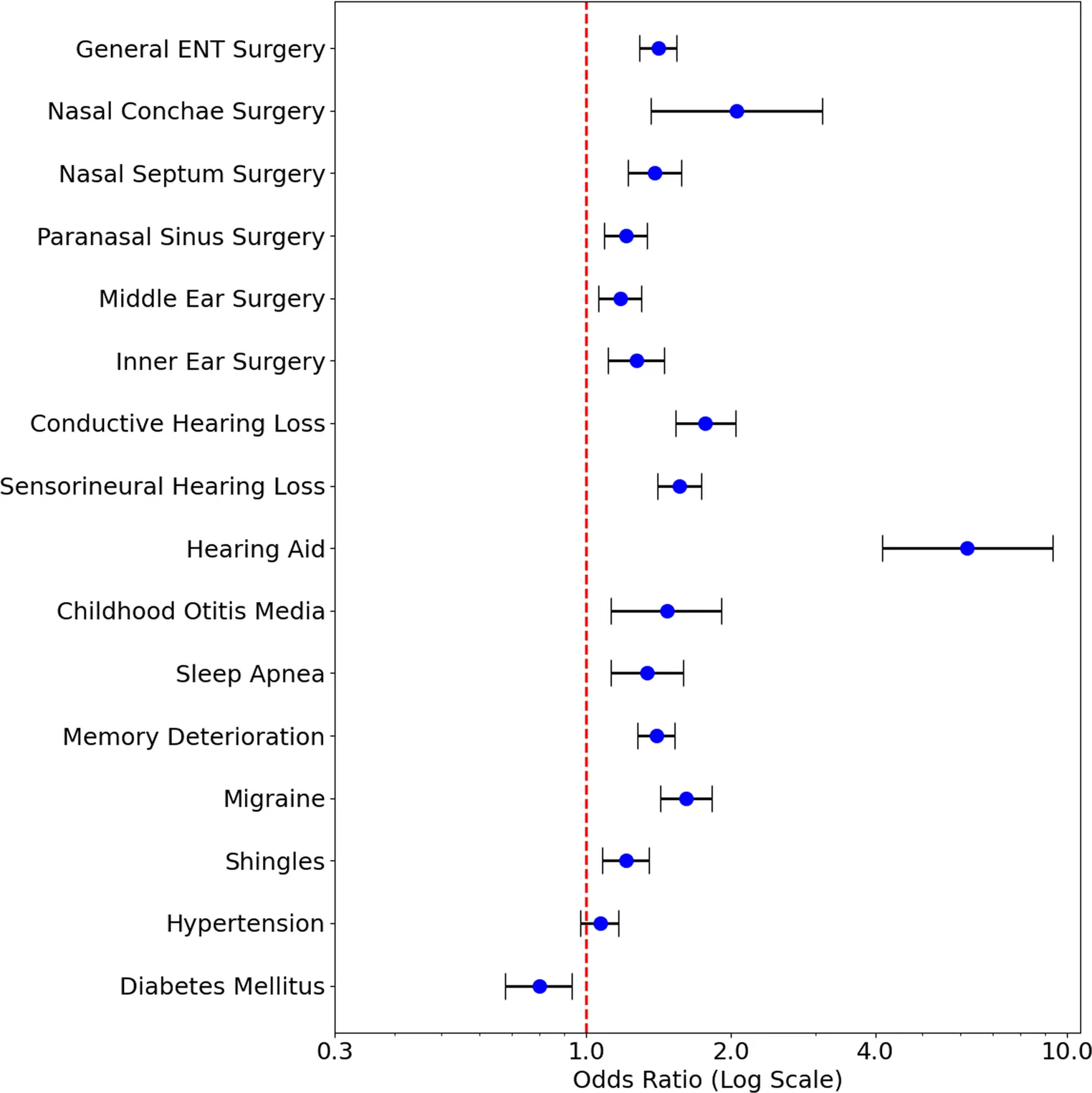
Odds ratios with 95% confidence intervals for comorbidities

**Fig. 3:**
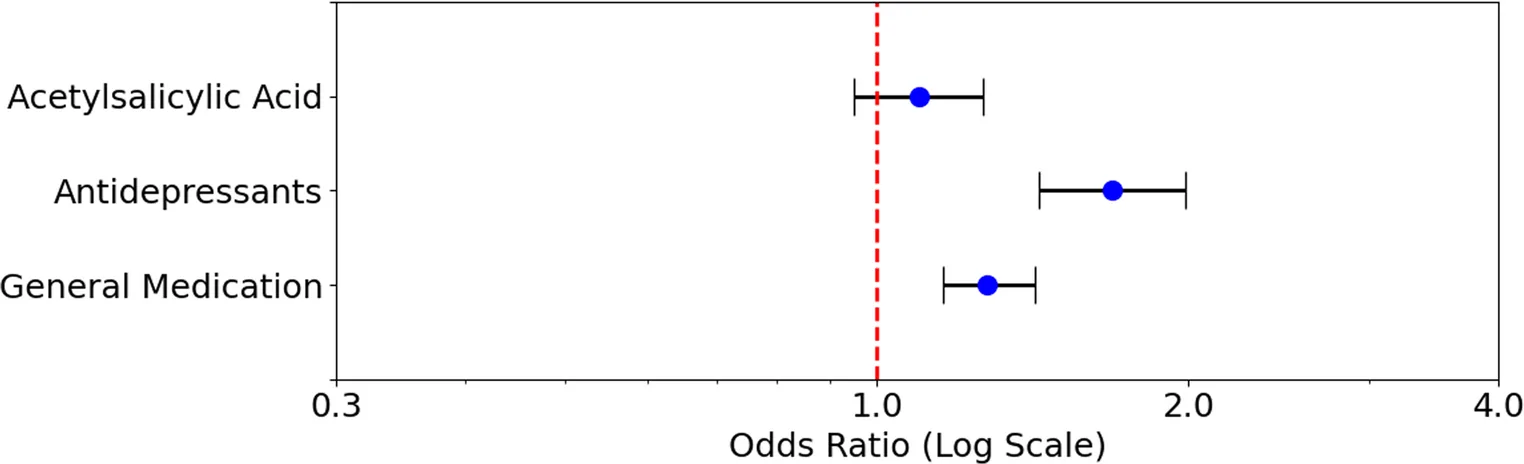
Odds ratios with 95% confidence intervals for medication

**Fig. 4:**
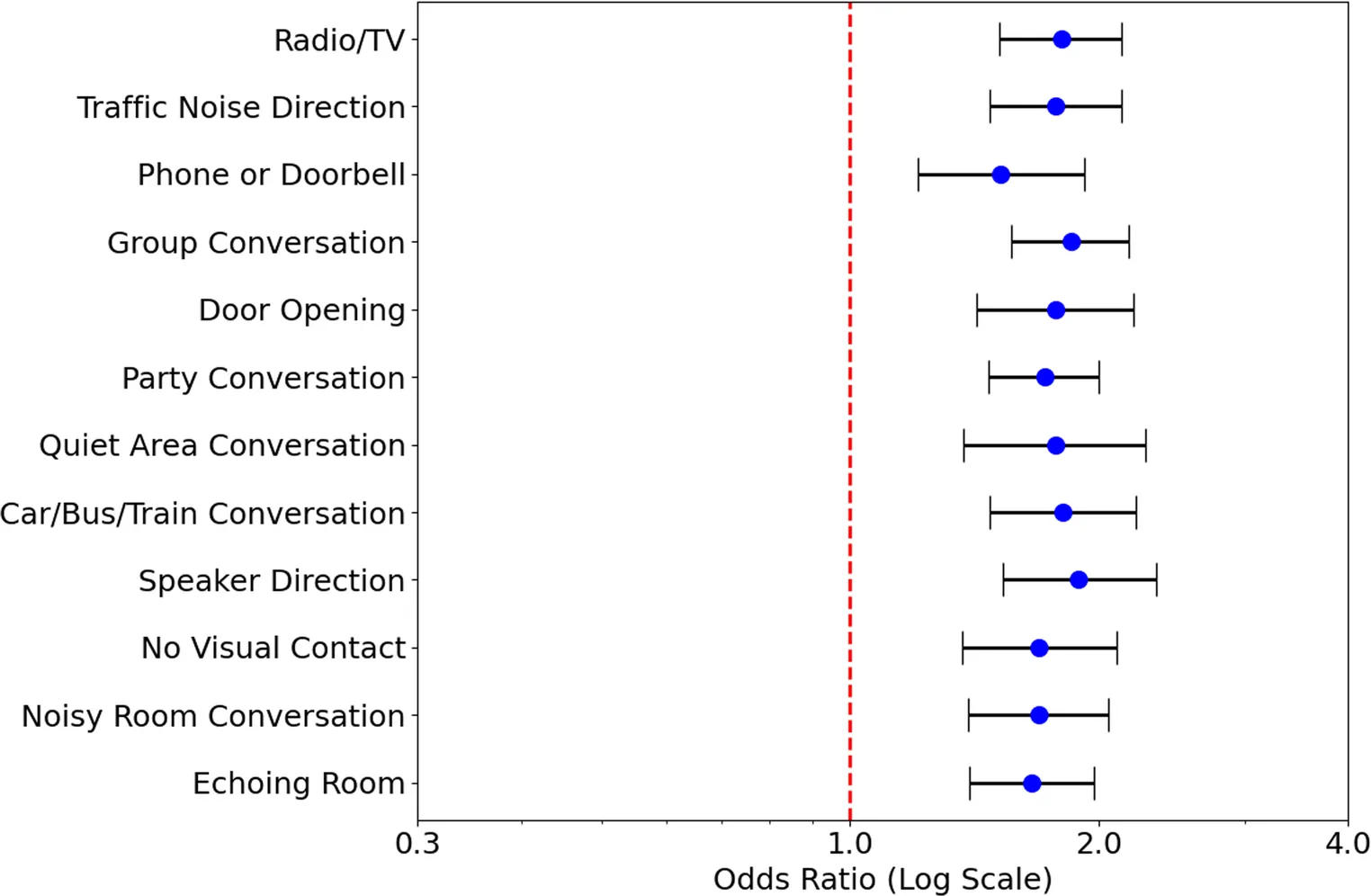
Odds ratios with 95% confidence intervals for subjective hearing problems

The results are organized into eight thematic categories:

1. demographics and lifestyle (Table 1; Fig. 1).
2. comorbidities (Table 2; Fig.2).
3. medication (Table 3; Fig.3).
4. subjective hearing problems (Table 4; Fig. 4).
5. subjective hearing problems with hearing aid (Supplementary material).
6. social effects of hearing problems (Table 5, Supplementary material - Fig. 6).
7. noise in living environment (Table 6, Supplementary material - Fig. 7).
8. noise exposure at work (Supplementary material).

Questions from categories 4, 6, and 8 were answered by a subset of 647 participants who reported hearing problems. Category 5 was answered by an even smaller subset of 107 patients who additionally reported hearing problems while using hearing aids.

The main analysis focuses on associations assessed to be clinically relevant. Full results for additional predictors not reported in the main paper, as well as unadjusted logistic regression models, are provided in the Supplementary Materials (Tables 7, 8, 9, 10, 11, 12, 13, 14, 15, 16, 17, 18, 19 and 20). The local ethics committee of the Landesaerztekammer Hamburg (State of Hamburg Chamber of Medical Practitioners, PV5131) did not have objections against the conduct of the Hamburg City Health Study (HCHS). The study has been registered at ClinicalTrial.gov (NCT03934957), and it is conducted in agreement with the Good Epidemiological Practice (GEP), and the Declaration of Helsinki [34]. All participants of HCHS gave their written informed consent.

## Results

Data from 14,355 participants in the Hamburg City Health Study (HCHS) were available for the main analysis presented in this article. Based on responses to the structured questionnaire, the lifetime self-reported physician-diagnosed prevalence of tinnitus was 19,3% (*n* = 2,765, Table 1). Mean age in the tinnitus group was slightly higher (Mean: 61.0) than in the non-tinnitus group (Mean: 60.5) in the total cohort (*p* = 0.006; Table 1). The structured HCHS questionnaires provided data for analysis of following categories.

**Table 1:** Statistical analysis of demographic and lifestyle factors using age- and sex- adjusted logistic regression

Variable	No Tinnitus	Tinnitus	p-value	Odds Ratio
Age	N = 11590	N = 2765		
60.6 [60.5, 60.7]	60.5 [60.3, 60.7]	61.0 [60.7, 61.3]	0.006*	1.01*
Sex	N = 11590	N = 2765		
Male	5693 (49.1%)	1463 (52.9%)		
Female	5897 (50.9%)	1302 (47.1%)	< 0.001*	0.86*
Employment Status	N = 10834	N = 2579		
Full-time	4753 (43.9%)	1136 (44.0%)	0.379	1.05
Part-time	1732 (16.0%)	328 (12.7%)	0.002	0.81
Partial retirement	131 (1.2%)	35 (1.4%)	0.639	1.09
Minijob	387 (3.6%)	98 (3.8%)	0.546	1.07
Irregularly employed	117 (1.1%)	34 (1.3%)	0.384	1.19
Not employed	3714 (34.3%)	948 (36.8%)	0.259	1.07
Occupational status if not in paid work	N = 3624	N = 930		
Retiree	3056 (84.3%)	779 (83.8%)	0.624	1.06
Unemployed	168 (4.6%)	40 (4.3%)	0.258	0.8
Permanently unable to work	66 (1.8%)	31 (3.3%)	0.013	1.77
Homemaker	220 (6.1%)	41 (4.4%)	0.024	0.66
Other	111 (3.1%)	35 (3.8%)	0.447	1.17
Occupational group	N = 10590	N = 2540		
Academic	656 (6.2%)	120 (4.7%)	0.003	0.74
Self-employed	1241 (11.7%)	268 (10.6%)	0.043	0.87
Civil servant	874 (8.3%)	275 (10.8%)	< 0.001	1.32
Employee	7017 (66.3%)	1698 (66.9%)	0.158	1.07
Laborer	802 (7.6%)	179 (7.0%)	0.17	0.89
Ever worked night-shifts?	N = 10708	N = 2569		
No	8837 (82.5%)	2069 (80.5%)		
Yes	1871 (17.5%)	500 (19.5%)	0.029	1.13

*Analysis for age and sex was done without any adjustments

### Demographics and lifestyle

**Fig. 5:**
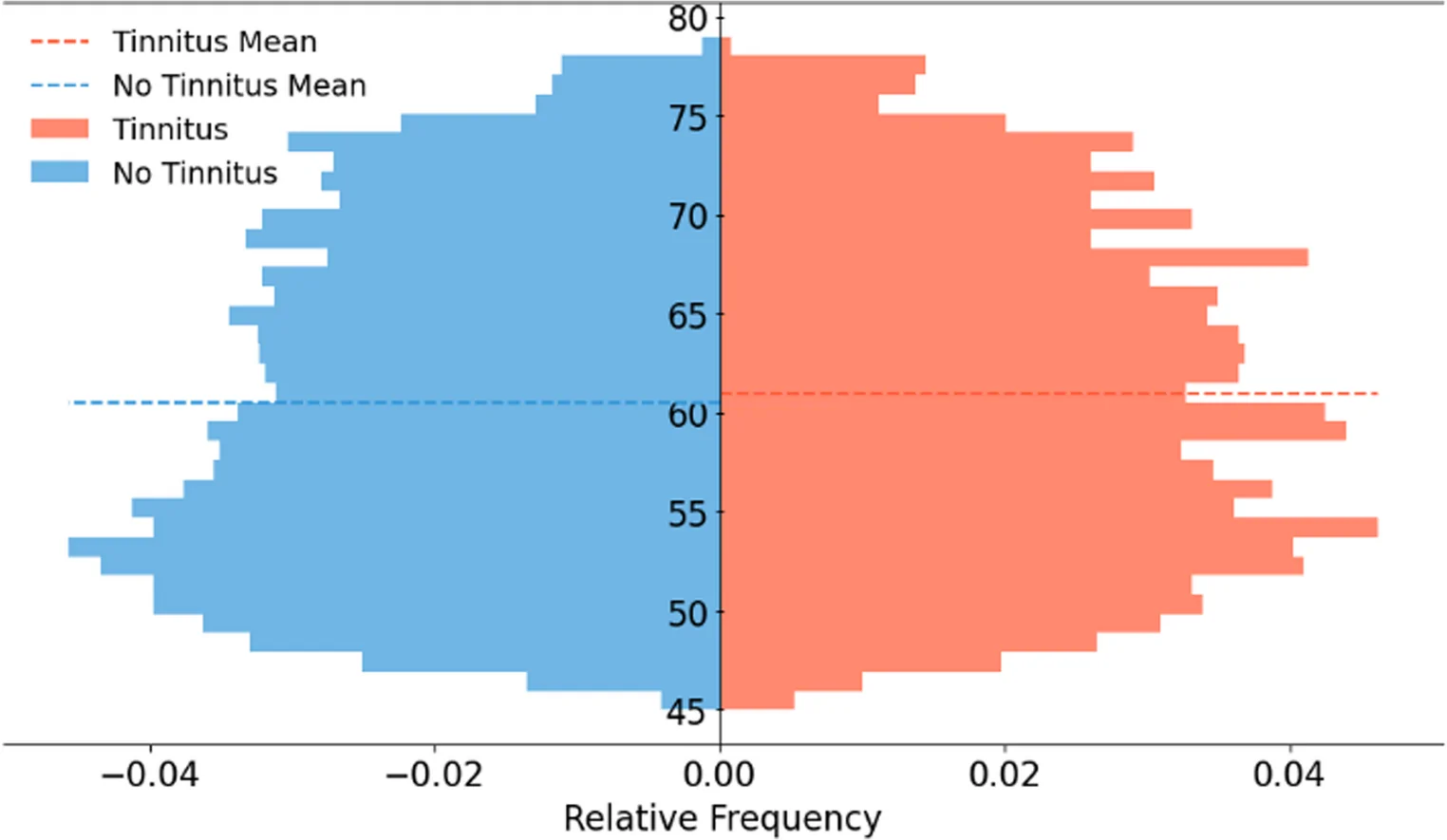
Age distribution of the full cohort

In the total cohort of 14,355 participants, there was no difference in sex distribution, with 49.9% (*n* = 7,156) male and 50.1% (*n* = 7,199) female participants (Fig. 5). However, within the tinnitus group, a higher proportion of participants were male (52.9%) compared to female (47.1%), indicating a positive association between tinnitus prevalence and male sex (Table 1).

Regarding employment status, the proportion of participants working part-time was higher in the non-tinnitus group (16.0%) compared to the tinnitus-group (12.7%). No differences were observed between groups for full-time employment or overall unemployment. However, among unemployed individuals, the proportion of permanent disability to work was higher in the tinnitus group (*p* = 0.013; odds ratio 1.77). Moreover, there were more people quoting to be unemployed in the unadjusted analysis (*p* = 0.018; Table 13). Occupational analysis revealed that individuals in academic professions were less likely to report tinnitus (*p* = 0.003; OR = 0.74). In contrast, employment as a civil servant was positively associated with tinnitus (*p* < 0.001; OR = 1.32). A relevant association was found between tinnitus and a history of night-shift work. Both in the age- and sex-adjusted analysis as well as the unadjusted analysis more participants quoted to have ever worked night shifts (*p* = 0.019). Also no association was found between tinnitus and smoking status, marital status, income and housing type in the matched group analysis (Supplementary material; Table 7).

### Comorbidities

The proportion of participants reporting a history of surgery in the ear, nose, and throat (ENT) region was higher in the tinnitus group (*p* < 0.001; OR = 1.41). Specific ENT procedures, including nasal conchae surgery, nasal septum surgery, paranasal sinus surgery, middle ear surgery, and inner ear surgery, were all more prevalent among individuals with tinnitus (Table 2). In contrast, the prevalence of pharynx surgery and neck soft tissue surgery (Supplementary material Table 8) did not differ between the groups. Known ear-related conditions, such as conductive hearing loss, sensorineural hearing loss, and a history of frequent otitis media in childhood, were more common in the tinnitus group, as was the use of hearing aids (Table 2). Sleep apnea was also more prevalent among tinnitus sufferers (*p* < 0.001, OR = 1.34). Additionally, self-reported memory deterioration, migraines, and a history of shingles (herpes zoster) infection were all associated with tinnitus. Conversely, diabetes mellitus was more common in the non-tinnitus group (*p* = 0.005; OR = 0.80). An exploratory analysis identified a possible association between carotid stenosis and tinnitus (*p* = 0.042); however, given the large number of comparisons performed and the absence of correction for multiple testing, this finding should be interpreted with caution and requires confirmation in future studies. No difference in the prevalence of any other cardiovascular conditions such as hypertension (Table 2), as well as history of heart attack, coronary artery disease (Supplementary material, Table 8) was observed between the two groups. Also no difference was seen in neurological conditions like Parkinson, multiple sclerosis and history of stroke (Supplementary material, Table 8).

**Table 2:** Statistical analysis of comorbidities using age- and sex- adjusted logistic regression

Variable	No Tinnitus	Tinnitus	p-value	Odds ratio
Had surgery in ENT region	N = 11338	N = 2691		
No	8035 (70.9%)	1702 (63.2%)		
Yes	3303 (29.1%)	989 (36.8%)	< 0.001	1.41
Nasal conchae surgery	N = 11180	N = 2652		
No	11107 (99.3%)	2618 (98.7%)		
Yes	73 (0.7%)	34 (1.3%)	< 0.001	2.05
Nasal septum surgery	N = 11180	N = 2652		
No	10977 (98.2%)	2562 (96.6%)		
Yes	203 (1.8%)	90 (3.4%)	< 0.001	1.39
Paranasal sinus surgery	N = 11180	N = 2652		
No	11030 (98.7%)	2592 (97.7%)		
Yes	150 (1.3%)	60 (2.3%)	< 0.001	1.21
Middle ear surgery	N = 11180	N = 2652		
No	11154 (99.8%)	2636 (99.4%)		
Yes	26 (0.2%)	16 (0.6%)	0.002	1.18
Inner ear surgery	N = 11180	N = 2652		
No	11172 (99.9%)	2642 (99.6%)		
Yes	8 (0.1%)	10 (0.4%)	< 0.001	1.27
Conductive hearing loss	N = 2340	N = 452		
No	2321 (99.2%)	419 (92.7%)		
Yes	19 (0.8%)	33 (7.3%)	< 0.001	1.77
Sensorineural hearing loss	N = 2340	N = 452	< 0.001	1.56
No	2316 (99.0%)	413 (91.4%)		
Yes	24 (1.0%)	39 (8.6%)		
Hearing aid	N = 2444	N = 585		
No	2399 (98.2%)	526 (89.9%)		
Yes	45 (1.8%)	59 (10.1%)	< 0.001	6.21
Frequent otitis media as child	N = 2221	N = 518		
No	1954 (88.0%)	432 (83.4%)		
Yes	267 (12.0%)	86 (16.6%)	0.004	1.47
Sleep apnea	N = 11212	N = 2677		
No	10639 (94.9%)	2489 (93.0%)		
Yes	573 (5.1%)	188 (7.0%)	< 0.001	1.34
Memory deterioration	N = 10240	N = 2430		
No	5625 (54.9%)	1136 (46.7%)		
Yes	4615 (45.1%)	1294 (53.3%)	< 0.001	1.4
Migraine	N = 11419	N = 2711		
No	10156 (88.9%)	2282 (84.2%)		
Yes	1263 (11.1%)	429 (15.8%)	< 0.001	1.61
Shingles	N = 11380	N = 2725		
No	9675 (85.0%)	2244 (82.3%)		
Yes	1705 (15.0%)	481 (17.7%)	< 0.001	1.21
Hypertension	N = 11442	N = 2728		
No	7482 (65.4%)	1721 (63.1%)		
Yes	3960 (34.6%)	1007 (36.9%)	0.166	1.07
Diabetes mellitus	N = 10783	N = 2599		
No	9802 (90.9%)	2397 (92.2%)		
Yes	981 (9.1%)	202 (7.8%)	0.005	0.8

### Medication

Overall medication use was higher in the tinnitus group compared to the control group (*p* < 0.001 OR = 1.28) (Table 3). The use of antidepressants was particularly associated with tinnitus, showing a strong correlation (*p* < 0.001; OR = 1.69). In contrast, the use of other medications - including acetylsalicylic acid, diuretics, beta blockers, ACE inhibitors, and aminoglycosides - did not differ between groups. Detailed results for these medications are presented in the Supplementary Materials (Table 9).

**Table 3:** Statistical analysis of medication using age- and sex- adjusted logistic regression

Variable	No Tinnitus	Tinnitus	p-value	Odds ratio
Acetylsalicylic acid	N = 11150	N = 2683		
No	10098 (90.6%)	2391 (89.1%)		
Yes	1052 (9.4%)	292 (10.9%)	0.202	1.1
Antidepressants	N = 11150	N = 2683		
No	10574 (94.8%)	2464 (91.8%)		
Yes	576 (5.2%)	219 (8.2%)	< 0.001	1.69
Medications (general)	N = 11150	N = 2683		
No	3117 (28.0%)	624 (23.3%)		
Yes	8033 (72.0%)	2059 (76.7%)	< 0.001	1.28

### Subjective hearing problems

The questionnaire consists of 12 situation-specific items assessing hearing difficulties in various everyday contexts – such as understanding radio or television at normal volume, or following conversations in echoic environments. Responses were rated on a five-point Likert scale ranging from 1 („always“) to 5 („never“). Across all scenarios, participants in the tinnitus group reported greater hearing difficulties compared to the control group. For example, a higher proportion of participants with tinnitus indicated difficulty consistently understanding conversations in rooms with multiple persons (*p* < 0.001, OR = 1.85; Table 4).

**Table 4:** Statistical analysis of subjective hearing problems using age- and sex- adjusted logistic regression. Answers are given on a scale from 1 (Always) to 5 (Never)

Variable	No Tinnitus	Tinnitus	p-value	Odds ratio
Understand radio/TV at room volume?	N = 390	N = 235		
1	244 (62.6%)	85 (36.2%)		
2	93 (23.8%)	70 (29.8%)		
3	35 (9.0%)	42 (17.9%)		
4	10 (2.6%)	26 (11.1%)		
5	8 (2.1%)	12 (5.1%)	< 0.001	1.8
Sudden loud traffic noise, can immediately tell direction?	N = 385	N = 233		
1	240 (62.3%)	99 (42.5%)		
2	111 (28.8%)	74 (31.8%)		
3	22 (5.7%)	28 (12.0%)		
4	6 (1.6%)	19 (8.2%)		
5	6 (1.6%)	13 (5.6%)	< 0.001	1.77
Quiet room, can hear phone or doorbell?	N = 391	N = 236		
1	341 (87.2%)	182 (77.1%)		
2	34 (8.7%)	29 (12.3%)		
3	8 (2.0%)	10 (4.2%)		
4	6 (1.5%)	11 (4.7%)		
Room with multiple persons, can understand conversations?	N = 391	N = 235	< 0.001	1.85
1	182 (46.5%)	46 (19.6%)		
2	127 (32.5%)	83 (35.3%)		
3	54 (13.8%)	51 (21.7%)		
4	19 (4.9%)	43 (18.3%)		
5	9 (2.3%)	12 (5.1%)		
Quite room, can hear door opening?	N = 390	N = 236	< 0.001	1.77
1	308 (79.0%)	144 (61.0%)		
2	63 (16.2%)	52 (22.0%)		
3	10 (2.6%)	24 (10.2%)		
4	6 (1.5%)	12 (5.1%)		
5	3 (0.8%)	4 (1.7%)		
Party, can easily have conversations?	N = 388	N = 232	< 0.001	1.72
1	120 (30.9%)	31 (13.4%)		
2	150 (38.7%)	73 (31.5%)		
3	70 (18.0%)	48 (20.7%)		
4	34 (8.8%)	63 (27.2%)		
5	14 (3.6%)	17 (7.3%)		
Walk in quite area, can easily have conversation?	N = 389	N = 234	< 0.001	1.77
1	324 (83.3%)	157 (67.1%)		
2	50 (12.9%)	53 (22.6%)		
3	11 (2.8%)	15 (6.4%)		
4	2 (0.5%)	5 (2.1%)		
5	2 (0.5%)	4 (1.7%)		
Car/Bus/Train, can easily have conversation?	N = 390	N = 235	< 0.001	1.81
1	252 (64.6%)	98 (41.7%)		
2	105 (26.9%)	92 (39.1%)		
3	24 (6.2%)	22 (9.4%)		
4	6 (1.5%)	17 (7.2%)		
5	3 (0.8%)	6 (2.6%)		
Can immediately tell where someone is speaking from?	N = 388	N = 236	< 0.001	1.89
1	271 (69.8%)	116 (49.2%)		
2	100 (25.8%)	74 (31.4%)		
3	8 (2.1%)	27 (11.4%)		
4	5 (1.3%)	12 (5.1%)		
5	4 (1.0%)	7 (3.0%)		
Quite room, can talk to someone without looking at the person?	N = 383	N = 233	< 0.001	1.69
1	297 (77.5%)	136 (58.4%)		
2	63 (16.4%)	61 (26.2%)		
3	15 (3.9%)	21 (9.0%)		
4	4 (1.0%)	10 (4.3%)		
Room with keyboard noise, music, etc., can easily have conversation?	N = 383	N = 233	< 0.001	1.69
1	222 (58.0%)	91 (39.1%)		
2	115 (30.0%)	82 (35.2%)		
3	35 (9.1%)	41 (17.6%)		
4	9 (2.3%)	15 (6.4%)		
5	2 (0.5%)	4 (1.7%)		
Easily understand spoken words in echoing rooms?	N = 388	N = 231	< 0.001	1.66
1	158 (40.7%)	55 (23.8%)		
2	153 (39.4%)	89 (38.5%)		
3	51 (13.1%)	47 (20.3%)		
4	19 (4.9%)	28 (12.1%)		
5	7 (1.8%)	12 (5.2%)		

### Subjective hearing problems with hearing aid

These items assessed hearing difficulties in everyday contexts while wearing hearing aids – for example, understanding radio or television at normal volume or following conversations in echoic environments. Responses were rated on a five-point Likert scale ranging from 1 („always“) to 5 („never“). None of the items showed a difference between the tinnitus and non-tinnitus groups (Supplementary material, Table 10).

### Social effects of hearing problems

Items addressed issues such as whether hearing loss interfered with interpersonal interactions or led participants to avoid group conversations. Responses were recorded on an eleven-point Likert scale ranging from 1 („never“) to 11 („always“). Again across all items, participants in the tinnitus group reported significantly greater social difficulties and a higher perceived impact of hearing problems on social interactions. For instance, a significantly greater proportion of participants with tinnitus reported that others find it difficult to engage in conversation with them (*p* < 0.001, OR = 1.25; Table 5).

**Table 5:** Statistical analysis of social effects of hearing problems using age- and sex- adjusted logistic regression. Answers are given on a scale from 1 (Never) to 11 (Always)

Variable	No Tinnitus	Tinnitus	p-value	Odds ratio
Hearing loss affects interactions with others?	N = 388	N = 237		
1	191 (49.2%)	44 (18.6%)		
2	35 (9.0%)	23 (9.7%)		
3	45 (11.6%)	30 (12.7%)		
4	4 (1.0%)	8 (3.4%)		
5	25 (6.4%)	11 (4.6%)		
6	51 (13.1%)	55 (23.2%)		
7	14 (3.6%)	17 (7.2%)		
8	3 (0.8%)	12 (5.1%)		
9	13 (3.4%)	24 (10.1%)		
10	4 (1.0%)	8 (3.4%)		
11	3 (0.8%)	5 (2.1%)	< 0.001	1.28
Avoid meetings because of difficulties following conversations?	N = 388	N = 236		
1	308 (79.4%)	139 (58.9%)		
2	19 (4.9%)	17 (7.2%)		
3	26 (6.7%)	23 (9.7%)		
4	4 (1.0%)	6 (2.5%)		
5	6 (1.5%)	3 (1.3%)		
6	14 (3.6%)	30 (12.7%)		
7	4 (1.0%)	7 (3.0%)		
8	2 (0.5%)	1 (0.4%)		
9	3 (0.8%)	8 (3.4%)		
10	2 (0.5%)	2 (0.8%)		
11	0 (0.0%)	0 (0.0%)	< 0.001	1.26
Feel like people ignore you because of hearing problems?	N = 388	N = 237		
1	349 (89.9%)	182 (76.8%)		
2	11 (2.8%)	14 (5.9%)		
3	15 (3.9%)	21 (8.9%)		
4	2 (0.5%)	5 (2.1%)		
5	2 (0.5%)	1 (0.4%)		
6	5 (1.3%)	12 (5.1%)		
7	1 (0.3%)	1 (0.4%)		
8	0 (0.0%)	0 (0.0%)		
9	3 (0.8%)	1 (0.4%)		
10	0 (0.0%)	0 (0.0%)		
Feel like others find it difficult to hold conversation with you?	N = 388	N = 237		
1	276 (71.1%)	118 (49.8%)		
2	38 (9.8%)	28 (11.8%)		
3	37 (9.5%)	40 (16.9%)		
4	4 (1.0%)	8 (3.4%)		
5	9 (2.3%)	9 (3.8%)		
6	15 (3.9%)	25 (10.5%)		
7	4 (1.0%)	3 (1.3%)		
8	1 (0.3%)	2 (0.8%)		
9	3 (0.8%)	4 (1.7%)		
10	0 (0.0%)	0 (0.0%)		
11	1 (0.3%)	0 (0.0%)	< 0.001	1.25
Feel excluded because of hearing problems?	N = 388	N = 237		
1	299 (77.1%)	133 (56.1%)		
2	21 (5.4%)	24 (10.1%)		
3	28 (7.2%)	34 (14.3%)		
4	6 (1.5%)	6 (2.5%)		
5	8 (2.1%)	5 (2.1%)		
6	17 (4.4%)	20 (8.4%)		
7	7 (1.8%)	5 (2.1%)		
8	0 (0.0%)	4 (1.7%)		
9	2 (0.5%)	5 (2.1%)		
10	0 (0.0%)	1 (0.4%)		
11	0 (0.0%)	0 (0.0%)	< 0.001	1.24
Hesitate to meet new people because of hearing problems?	N = 386	N = 237		
1	356 (92.2%)	189 (79.7%)		
2	5 (1.3%)	8 (3.4%)		
3	11 (2.8%)	13 (5.5%)		
4	5 (1.3%)	7 (3.0%)		
5	2 (0.5%)	4 (1.7%)		
6	4 (1.0%)	10 (4.2%)		
7	1 (0.3%)	1 (0.4%)		
8	1 (0.3%)	1 (0.4%)		
9	1 (0.3%)	4 (1.7%)		
10	0 (0.0%)	0 (0.0%)		
11	0 (0.0%)	0 (0.0%)	< 0.001	1.32
Avoid group conversations, because of fear to give inappropriate answers?	N = 387	N = 237	< 0.001	1.26
1	307 (79.3%)	141 (59.5%)		
2	22 (5.7%)	18 (7.6%)		
3	27 (7.0%)	28 (11.8%)		
4	3 (0.8%)	5 (2.1%)		
5	4 (1.0%)	5 (2.1%)		
6	15 (3.9%)	25 (10.5%)		
7	5 (1.3%)	2 (0.8%)		
8	0 (0.0%)	5 (2.1%)		
9	3 (0.8%)	5 (2.1%)		
10	0 (0.0%)	2 (0.8%)		
Hearing problems affect self-confidence?	N = 387	N = 236		
1	322 (83.2%)	150 (63.6%)		
2	20 (5.2%)	21 (8.9%)		
3	16 (4.1%)	25 (10.6%)		
4	9 (2.3%)	2 (0.8%)		
5	5 (1.3%)	8 (3.4%)		
6	9 (2.3%)	19 (8.1%)		
7	2 (0.5%)	3 (1.3%)		
8	1 (0.3%)	3 (1.3%)		
9	2 (0.5%)	4 (1.7%)		
10	0 (0.0%)	1 (0.4%)		
11	1 (0.3%)	0 (0.0%)	< 0.001	1.29
Feel inferior because of hearing problems?	N = 387	N = 237		
1	344 (88.9%)	174 (73.4%)		
2	12 (3.1%)	20 (8.4%)		
3	12 (3.1%)	23 (9.7%)		
4	4 (1.0%)	5 (2.1%)		
5	7 (1.8%)	4 (1.7%)		
6	6 (1.6%)	7 (3.0%)		
7	0 (0.0%)	0 (0.0%)		
8	1 (0.3%)	2 (0.8%)		
9	1 (0.3%)	1 (0.4%)		
10	0 (0.0%)	1 (0.4%)		
11	0 (0.0%)	0 (0.0%)	< 0.001	1.27
Feel sad or frustrated when not able participate in conversation because of hearing problems?	N = 387	N = 237		
1	284 (73.4%)	125 (52.7%)		
2	26 (6.7%)	23 (9.7%)		
3	23 (5.9%)	25 (10.5%)		
4	13 (3.4%)	6 (2.5%)		
5	12 (3.1%)	8 (3.4%)		
6	18 (4.7%)	33 (13.9%)		
7	6 (1.6%)	6 (2.5%)		
8	2 (0.5%)	2 (0.8%)		
9	2 (0.5%)	4 (1.7%)		
10	1 (0.3%)	3 (1.3%)		
11	0 (0.0%)	2 (0.8%)	< 0.001	1.24

### Noise in living environment

All HCHS participants responded to questions regarding noise in their living environment, which included situation-specific items such as whether they felt disturbed by human noise or air traffic noise. Responses were recorded on a four-point Likert scale ranging from 0 („not at all“) to 3 („very much“). All of the items demonstrated a relevant difference between the tinnitus and non-tinnitus groups (Table 6), indicating that individuals with tinnitus reported higher levels of noise disturbance in their living environment.

**Table 6:** Statistical analysis of disturbance by noise in the living environment using age- and sex- adjusted logistic regression. Answers are given on a scale from 0 (Not at all) to 3 (Very much)

Variable	No Tinnitus	Tinnitus	p-value	Odds ratio
Feel disturbed by noise at home - weekdays, daytime	N = 11425	N = 2722		
0	6794 (59.5%)	1501 (55.1%)		
1	3853 (33.7%)	988 (36.3%)		
2	626 (5.5%)	177 (6.5%)		
3	152 (1.3%)	56 (2.1%)	< 0.001	1.16
Feel disturbed by noise at home - weekends, daytime	N = 11399	N = 2717		
0	7008 (61.5%)	1554 (57.2%)		
1	3735 (32.8%)	958 (35.3%)		
2	542 (4.8%)	161 (5.9%)		
3	114 (1.0%)	44 (1.6%)	< 0.001	1.18
Feel disturbed by noise at home - nighttime	N = 11399	N = 2712		
0	8553 (75.0%)	1968 (72.6%)		
1	2417 (21.2%)	618 (22.8%)		
2	321 (2.8%)	94 (3.5%)		
3	108 (0.9%)	32 (1.2%)	0.001	1.12
Feel disturbed by road traffic noise	N = 11280	N = 2696		
0	6874 (60.9%)	1560 (57.9%)		
1	3469 (30.8%)	874 (32.4%)		
2	661 (5.9%)	186 (6.9%)		
3	276 (2.4%)	76 (2.8%)	< 0.001	1.1
Feel disturbed by air traffic noise	N = 11211	N = 2690		
0	7629 (68.0%)	1727 (64.2%)		
1	2858 (25.5%)	734 (27.3%)		
2	556 (5.0%)	178 (6.6%)		
3	168 (1.5%)	51 (1.9%)	< 0.001	1.14
Feel disturbed by human noise - outdoors	N = 10995	N = 2627		
0	7445 (67.7%)	1704 (64.9%)		
1	3118 (28.4%)	790 (30.1%)		
2	354 (3.2%)	101 (3.8%)		
3	78 (0.7%)	32 (1.2%)	< 0.001	1.16
Feel disturbed by human noise - indoors	N = 11207	N = 2684		
0	8132 (72.6%)	1861 (69.3%)		
1	2699 (24.1%)	703 (26.2%)		
2	312 (2.8%)	90 (3.4%)		
3	64 (0.6%)	30 (1.1%)	< 0.001	1.18
Feel disturbed by miscellaneous noise	N = 11070	N = 2628		
0	8268 (74.7%)	1819 (69.2%)		
1	2533 (22.9%)	689 (26.2%)		
2	206 (1.9%)	91 (3.5%)		
3	63 (0.6%)	29 (1.1%)	< 0.001	1.31

### Noise exposure at work

Items addressed scenarios such as the ability to perceive warning signals or machine noise, and the frequency of work-related phone calls. Responses were rated on a five-point Likert scale ranging from 1 („always“) to 5 („never“). Only specific hygiene requirements, meaning conditions using for example gloves or having dirty hands working in a car workshop, was possibly associated with tinnitus (*p* = 0.028). Across all scenarios, no relevant differences were observed between the tinnitus and non-tinnitus groups (Supplementary material, Table 12).

## Discussion

We investigated the prevalence and potential demographic, socioeconomic, medical and environmental correlates of tinnitus within urban areas – the Hamburg City Health Study (HCHS), a large population-based cohort in Western Europe. Our findings confirm that tinnitus is a multifactorial condition with relevant associations across a range of factors, including sex, comorbid medical conditions, medication use, subjective hearing difficulties, social participation, and environmental noise exposure. Conversely, no relevant associations were found for several commonly discussed factors, such as alcohol consumption, marital status, and income.

### Prevalence and sociodemographic influences

The lifetime self-reported physician-diagnosed prevalence of tinnitus in our sample was 19.3%, aligning with previous estimates ranging from 8% to 25% in the general population in the United States [12] and 8.7% to 28.3% in Europe [13] depending on the country and methodology employed. Differences between studies may reflect variation in tinnitus definitions (e.g., current tinnitus versus lifetime physician-diagnosed tinnitus), age structure, urbanization, environmental noise exposure, healthcare access, and data collection methods. Compared to the global pooled prevalence of 14.4% [11] and prevalence of 11.9% in Germany [13] the lifetime self-reported physician-diagnosed prevalence in the urban area of Hamburg (HCHS) was higher. This may be affected by the noise exposure in the urban area and the self-reported questionnaire, which did not graduate between tinnitus types and duration. The comparatively high prevalence observed in the HCHS may also partly be explained by the older age of the cohort. Similar variability in tinnitus prevalence across populations has been highlighted in recent epidemiological investigations, emphasizing the importance of considering methodological and population-specific factors when comparing prevalence estimates between cohorts [35]. In agreement with prior studies [15–18], we observed a higher prevalence among male participants, although the literature remains inconclusive, with some studies reporting no sex difference or even higher rates in females [11, 19–22]. This discrepancy may reflect cultural, occupational, or methodological differences between study populations.

Age was also positively associated with tinnitus, with affected individuals being slightly older on average [11]. This finding is consistent with the well-established links between age-related auditory degeneration and tinnitus and suggests cumulative lifetime noise exposure or comorbid conditions as possible mediators.

### Lifestyle and socioeconomic factors

Socioeconomic variables revealed differing results. Contrary to prior research suggesting strong links between low socioeconomic status and tinnitus burden [23–26], we found no significant association with education level or overall employment status in the age- and sex-adjusted analysis. However, permanent unemployment and night-shift work were more prevalent in the tinnitus group, and individuals in academic professions were less likely to report tinnitus, while those employed as civil servants showed a higher prevalence. These patterns suggest that occupational context – rather than employment per se – may be a more sensitive indicator of tinnitus risk, potentially due to differences in job-related stress, noise exposure, or coping resources.

Importantly, our findings challenge oversimplified assumptions that lower socioeconomic status uniformly increases tinnitus prevalence.

### Medical comorbidities

Lifetime self-reported physician-diagnosed tinnitus was strongly associated with a range of otologic and non-otologic comorbidities. Participants with a history of ENT surgeries – especially those involving the nasal conchae, septum, sinuses, or inner ear – had higher odds of reporting tinnitus. This supports the established link between peripheral auditory system alterations and tinnitus development.

Hearing-related conditions, including sensorineural and conductive hearing loss and a history of recurrent otitis media, were more prevalent in the tinnitus group. These associations underscore the central role of auditory dysfunction in tinnitus pathogenesis and symptom severity.

An exploratory association between carotid stenosis and tinnitus was observed. Given the exploratory nature of the analyses and the lack of adjustment for multiple testing, this result should be regarded as hypothesis-generating rather than evidence of an independent relationship. Association with carotid stenosis suggests a possible vascular contribution to its pathophysiology. Hemodynamic changes such as turbulent blood flow or reduced cochlear perfusion may underlie this relationship, particularly in cases of pulsatile tinnitus. However, other cardiovascular conditions such as hypertension, history of heart attack and coronary artery disease showed no difference between the two groups. Therefore, the presence of carotid stenosis should not be interpreted as a direct causal factor without comprehensive otologic evaluation.

Unexpectedly, diabetes mellitus was negatively associated with tinnitus. This finding contrasts with some previous reports and may reflect unmeasured confounding factors or differences in comorbidity patterns, medication use, or metabolic control within this cohort. Future research is needed to clarify the nature of this relationship.

Use of antidepressants was markedly higher among tinnitus sufferers, aligning with previous research indicating increased rates of depression and anxiety in this population [1–4]. The results point toward a potentially bidirectional relationship, in which tinnitus and depressive symptoms may mutually reinforce each other, though the temporal and causal dynamics remain unclear.

### Hearing problems and noise exposure

Participants with lifetime self-reported physician-diagnosed tinnitus reported greater subjective hearing difficulties across a wide range of everyday scenarios. However, among the subgroup of hearing-aid users, no significant differences were observed between participants with and without tinnitus. This finding may suggest that hearing aids partially mitigate perceived hearing difficulties, although the relatively small sample size of hearing-aid users limits definitive conclusions.

Social consequences of hearing problems were also more pronounced in the tinnitus group. Respondents reported greater difficulties in group communication and perceived higher levels of social withdrawal and conversational challenges. These findings are consistent with prior literature and reinforce the broader psychosocial impact of tinnitus beyond the auditory domain.

A novel contribution of this analysis lies in its examination of environmental noise. Participants with self-reported lifetime physician-diagnosed tinnitus reported higher levels of perceived noise disturbance in their living environment, including human and air traffic noise. Importantly, the questionnaire assessed subjective disturbance rather than objectively measured environmental noise exposure. Therefore, the observed associations may reflect greater environmental noise exposure, increased sensitivity to sound, heightened awareness of environmental stimuli, or other perceptual factors. The present data do not allow these possibilities to be distinguished. Interestingly, no association was observed between tinnitus and workplace noise exposure. This may be due to reporting bias, retrospective assessment limitations, or protective regulations in occupational settings.

### Future potential of artificial intelligence in tinnitus research

An emerging area of interest in tinnitus research is the application of artificial intelligence (AI) and large language models (LLMs) to support both research and clinical care. The increasing number of demographic, audiological, clinical, psychological, lifestyle, and environmental variables associated with tinnitus makes the condition particularly suitable for data-driven prediction approaches. This was shown by the over the time improving and unexpectedly good results of an AI on tinnitus questions [36]. Future AI-based models may help identify individuals at increased risk of developing tinnitus, predict symptom burden, and improve patient stratification for targeted interventions. In clinical practice, AI-assisted tools and LLMs may support individualized patient education, symptom monitoring, and multidisciplinary management by providing accessible information and facilitating communication between patients and healthcare professionals. Although these technologies require careful validation and should complement rather than replace clinical expertise, they represent a promising avenue for advancing personalized tinnitus care and research.

### Limitations

Following limitations warrant consideration. First, the regression analyses were exploratory and based on separate age- and sex-adjusted logistic regression models for each variable. Accordingly, the reported odds ratios reflect adjusted association rather than mutually independent effects. Given the large number of potentially interrelated variables included in the analyses, residual confounding and collinearity cannot be excluded. Therefore, the findings should be interpreted as exploratory associations that require further confirmation in more comprehensive multivariable models.

Second, tinnitus was assessed via self-report questionnaires (“Have you ever been diagnosed with tinnitus by a doctor?”) without audiometric confirmation, which may introduce reporting bias or misclassification. Third, the cross-sectional, retrospective design precludes causal inference. Fourth, certain sub-analyses (e.g., those involving hearing-aid users or specific occupational groups) were based on relatively small sample sizes, limiting statistical power.

Moreover, potentially relevant factors such as tinnitus duration, severity, or associated distress were not assessed, which could have offered further insight into the heterogeneity of the condition. Finally, our findings are based on an urban, Western European cohort that is more educated and healthier which may limit generalizability to rural or non-European populations.

### Strenghts

This study has several important strengths. First, it is based on a large, well-characterized, population-based cohort with more than 14,000 participants, providing substantial statistical power to detect associations across a wide range of variables. Second, the analysis includes an extensive set of demographic, socioeconomic, lifestyle, medical, hearing-related, and environmental factors, allowing for a comprehensive assessment of potential correlates of tinnitus within a single study population. Third, data were collected using standardized, structured questionnaires within the Hamburg City Health Study, ensuring consistency in measurement across participants. Fourth, the study examines tinnitus within a contemporary European urban population, contributing valuable evidence from a setting that is underrepresented in the existing literature. Finally, the use of age- and sex-adjusted regression models enables consistent comparison of associations across multiple domains.

## Conclusion

In conclusion, lifetime self-reported physician-diagnosed tinnitus affected nearly one in five participants in this large urban cohort, underscoring its relevance as a major public health concern. Our findings reinforce the concept of tinnitus as a multifactorial condition in which auditory dysfunction, medical comorbidities, psychological factors, and environmental influences interact to shape symptom occurrence and burden.

As predicted, this large-scale study found a slightly higher prevalence of tinnitus than currently reported in review articles, and we attribute this to the urban population. The risk factors identified point in particular to medical influences on tinnitus burden. Recent research and the data of our study emphasizes that, beyond auditory pathology, several modifiable lifestyle factors contribute to both the development of tinnitus. Beyond confirming established associations with hearing loss and ENT-related conditions, this study highlights the broader impact of tinnitus on social participation and everyday functioning, as well as its links with perceived environmental noise and specific occupational contexts. These results suggest that tinnitus should not be viewed solely as an isolated auditory symptom but rather as a condition with substantial biopsychosocial dimensions.

The findings support the need for interdisciplinary care models that combine audiological assessment with psychological, social, and environmental interventions. They also emphasize the importance of early identification of hearing-related disorders and modifiable risk factors in populations at increased risk. Future longitudinal studies are needed to clarify causal pathways and to develop targeted prevention and treatment strategies that reduce both the auditory and non-auditory burden of tinnitus.

##  Supplementary material

Below is the link to the electronic supplementary material.

Supplementary Material 1
