# Supplementary material for: Tinnitus in urban settings: insights into lifestyle and medical factors from the Hamburg City Health Study
Source: Eur Arch Otorhinolaryngol. 2026 Sep 29;283(11):7169–87. doi: 10.1007/s00405-026-10518-z (PMC13748036; doi:10.1007/s00405-026-10518-z)
Supplement: Supplementary file 1 [file 405_2026_10518_MOESM1_ESM.pdf]

Supplementary
Figures

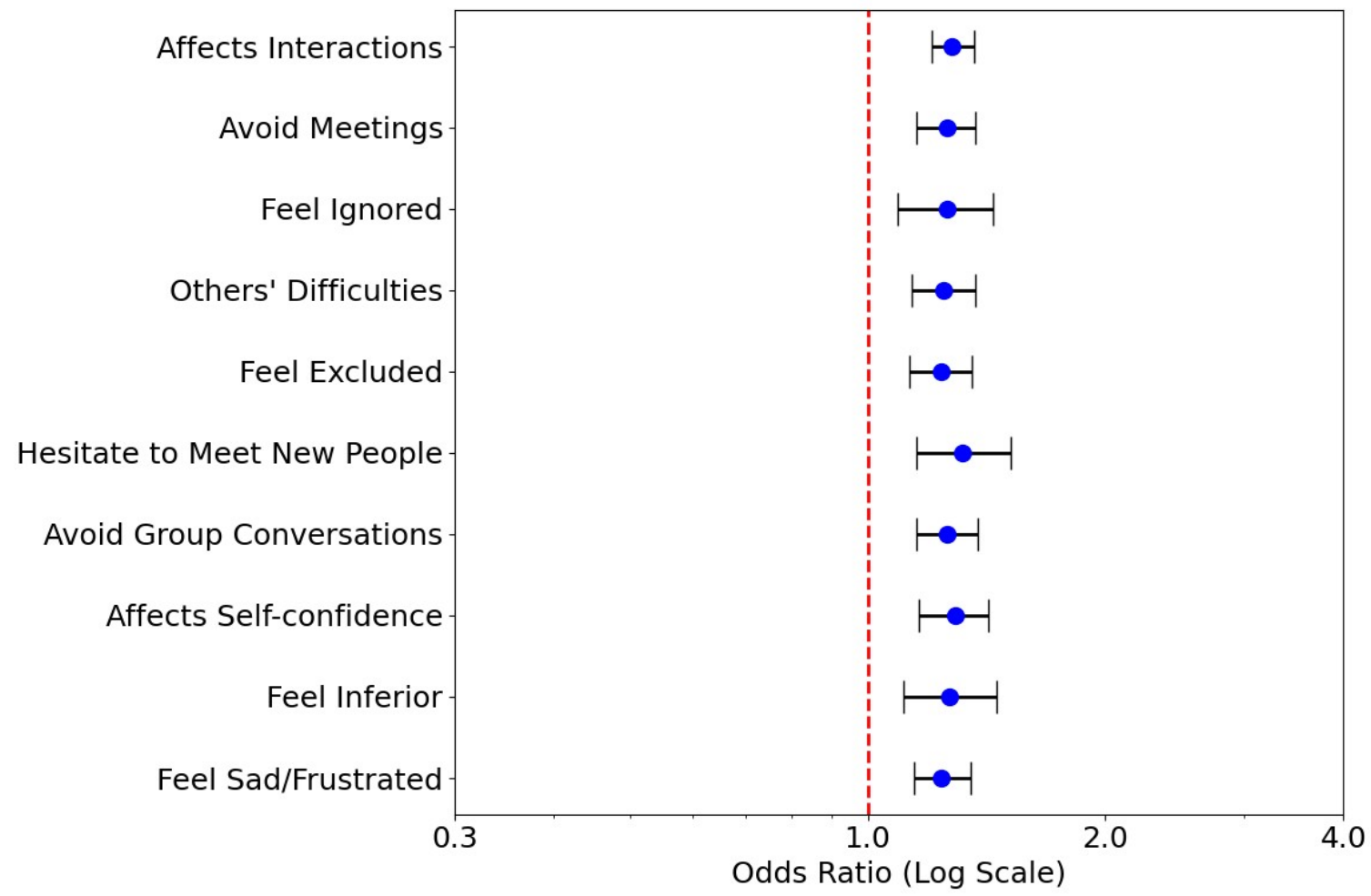

Figure 6: Odds ratios and 95% confidence intervals for social effects of hearing problems.

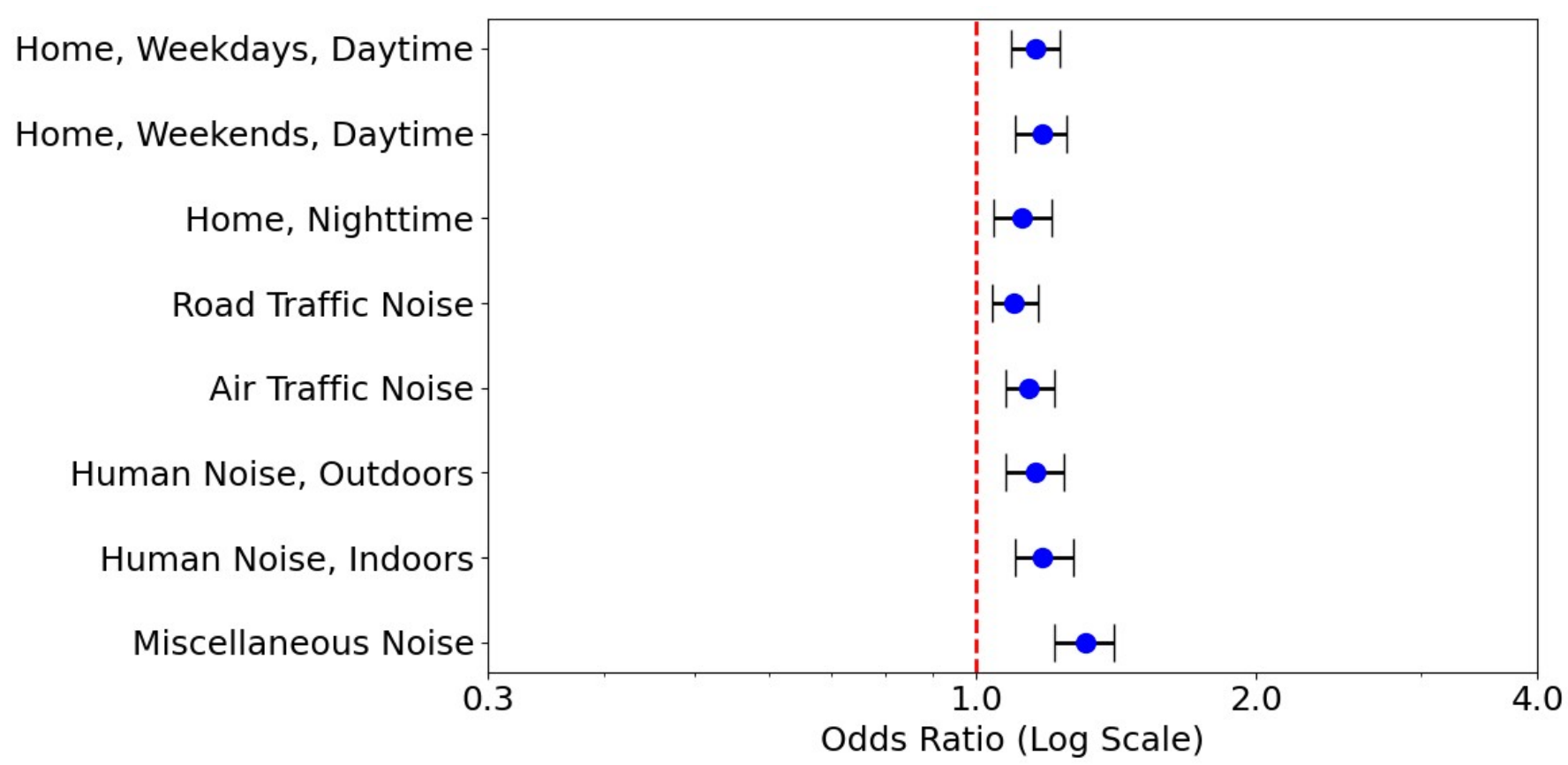

Figure 7: Odds ratios and 95% confidence intervals for disturbance by noise in the living environment.

Age- and sex- adjusted logistic regression

Table 7: Statistical analysis of the remaining demographic and lifestyle factors using age- and sex-adjusted logistic regression.

Variable	No Tinnitus	Tinnitus	p-value	Odds Ratio
Are you smoking?	N = 11533	N = 2752		
Yes	2410 (20.9%)	519 (18.9%)	0.037	0.89
No (min. 6 Month)	4646 (40.3%)	1214 (44.1%)	0.003	1.14
No (never)	4477 (38.8%)	1019 (37.0%)	0.188	0.94

Martial Status	N = 11417	N = 2721		
Married, living together	7223 (63.3%)	1725 (63.4%)	0.684	0.98
Married, living separately	308 (2.7%)	70 (2.6%)	0.83	0.97
Single	1752 (15.3%)	401 (14.7%)	0.781	0.98
Divorced	1504 (13.2%)	377 (13.9%)	0.226	1.08
Widowed	630 (5.5%)	148 (5.4%)	0.712	0.96
Committed Relationship				
No	N = 6962	N = 1670		
Yes, permanently living	2228 (32.0%)	544 (32.6%)	0.692	1.02
together	3783 (54.3%)	888 (53.2%)	0.414	0.96
Yes, partially living together	524 (7.5%)	117 (7.0%)	0.438	0.92
Yes, living separately	427 (6.1%)	121 (7.2%)	0.084	1.2
Since when employed? (year) 1993.8 [1993.5, 1994.1]	N = 6576 1993.9 [1993.6, 1994.2]	N = 1511 1993.3 [1992.6, 1993.9]	0.417	1.0
Working hours 34.9 [34.6, 35.2]	N = 5968 34.8 [34.5, 35.2]	N = 1356 35.2 [34.5, 35.8]	0.392	1.0
Ever worked full/part-time?	N = 2438	N = 664		
No	65 (2.7%)	20 (3.0%)		
Yes	2373 (97.3%)	644 (97.0%)	0.613	0.88
Ever been unemployed?	N = 11033	N = 2645		
No	5626 (51.0%)	1294 (48.9%)		
Yes	5407 (49.0%)	1351 (51.1%)	0.013	1.12
How many times unemployed? 1.8 [1.7, 1.8]	N = 4466 1.8 [1.7, 1.9]	N = 1106 1.8 [1.7, 1.8]	0.574	0.99
How long unemployed? (months) 17.8 [17.0, 18.7]	N = 4249 17.9 [16.9, 18.9]	N = 1049 17.5 [15.6, 19.4]	0.569	1.0
Currently unemployed?	N = 5165	N = 1302		
No	4899 (94.8%)	1225 (94.1%)		
Yes	266 (5.2%)	77 (5.9%)	0.202	1.19
Net income (1=<500€	N = 9353	N = 2234		
-17=8000€+)	485 (5.2%)	95 (4.3%)		
1	310 (3.3%)	50 (2.2%)		
2	550 (5.9%)	113 (5.1%)		
3	620 (6.6%)	157 (7.0%)		
4	726 (7.8%)	173 (7.7%)		
5	697 (7.5%)	172 (7.7%)		
6	797 (8.5%)	191 (8.5%)		
7	795 (8.5%)	197 (8.8%)		
8	714 (7.6%)	161 (7.2%)		
9	966 (10.3%)	245 (11.0%)		
10	752 (8.0%)	189 (8.5%)		
11	450 (4.8%)	134 (6.0%)		
12	359 (3.8%)	98 (4.4%)	0.157	1.01

13				
14	269 (2.9%)	69 (3.1%)		
15	310 (3.3%)	71 (3.2%)		
16	251 (2.7%)	54 (2.4%)		
17	302 (3.2%)	65 (2.9%)		
Household income (1=<500€ -17=8000€+)	N = 8861	N = 2117		
1	64 (0.7%)	9 (0.4%)		
2	63 (0.7%)	18 (0.9%)		
3	181 (2.0%)	42 (2.0%)		
4	245 (2.8%)	57 (2.7%)		
5	320 (3.6%)	65 (3.1%)		
6	307 (3.5%)	84 (4.0%)		
7	422 (4.8%)	115 (5.4%)		
8	481 (5.4%)	132 (6.2%)		
9	536 (6.0%)	122 (5.8%)		
10	905 (10.2%)	193 (9.1%)		
11	948 (10.7%)	239 (11.3%)		
12	778 (8.8%)	184 (8.7%)		
13	679 (7.7%)	164 (7.7%)		
14	627 (7.1%)	163 (7.7%)		
15	848 (9.6%)	207 (9.8%)		
16	758 (8.6%)	183 (8.6%)		
17	699 (7.9%)	140 (6.6%)	0.544	1.0
Housing type	N = 11478	N = 2733		
Detached single-family house	2391 (20.8%)	536 (19.6%)	0.12	0.92
Semi-detached house	1067 (9.3%)	260 (9.5%)	0.743	1.02
Terraced house	1343 (11.7%)	341 (12.5%)	0.274	1.07
Multi-family house	5964 (52.0%)	1439 (52.7%)	0.438	1.03
high-rise building	481 (4.2%)	100 (3.7%)	0.223	0.87
Other	232 (2.0%)	57 (2.1%)	0.81	1.04
Rent/ownership	N = 11461	N = 2734		
Rent	5565 (48.6%)	1330 (48.6%)	0.708	1.02
Ownership	5811 (50.7%)	1385 (50.7%)	0.739	0.99
Other	85 (0.7%)	19 (0.7%)	0.809	0.94

Table 8: Statistical analysis of the remaining comorbidities using age- and sex- adjusted logistic regression.

Variable	No Tinnitus	Tinnitus	p-value	Odds Ratio
Heart Attack	N = 11502	N = 2736		
No	11186 (97.3%)	2654 (97.0%)		
Yes	316 (2.7%)	82 (3.0%)	0.98	1.0

Coronary artery disease	N = 11251	N = 2660		
No	10760 (95.6%)	2519 (94.7%)		
Yes	491 (4.4%)	141 (5.3%)	0.197	1.14
Angia pectoris	N = 10610	N = 2529		
No	10235 (96.5%)	2421 (95.7%)		
Yes	375 (3.5%)	108 (4.3%)	0.117	1.19
Claudication	N = 11202	N = 2634		
No	10838 (96.8%)	2541 (96.5%)		
Yes	364 (3.2%)	93 (3.5%)	0.674	1.05
Carotid stenosis	N = 11169	N = 2636		
No	10736 (96.1%)	2505 (95.0%)		
Yes	433 (3.9%)	131 (5.0%)	0.042	1.23
External nose surgery	N = 11180	N = 2652		
No	11168 (99.9%)	2645 (99.7%)		
Yes	12 (0.1%)	7 (0.3%)	0.043	1.27
Outer ear surgery	N = 11180	N = 2652		
No	11156 (99.8%)	2644 (99.7%)		
Yes	24 (0.2%)	8 (0.3%)	0.361	1.08
Pharynx surgery via mouth	N = 11180	N = 2652		
No	10995 (98.3%)	2596 (97.9%)		
Yes	185 (1.7%)	56 (2.1%)	0.066	1.04
Pharynx surgery external	N = 11180	N = 2652		
No	11173 (99.9%)	2649 (99.9%)		
Yes	7 (0.1%)	3 (0.1%)	0.383	1.07
Larynx surgery via mouth	N = 11180	N = 2652		
No	11175 (100.0%)	2648 (99.8%)		
Yes	5 (0.0%)	4 (0.2%)	0.063	1.13
Larynx surgery external	N = 11180	N = 2652		
No	11178 (100.0%)	2650 (99.9%)		
Yes	2 (0.0%)	2 (0.1%)	0.139	1.14
Neck soft tissue surgery	N = 11180	N = 2652		
No	11158 (99.8%)	2648 (99.8%)		
Yes	22 (0.2%)	4 (0.2%)	0.671	0.98
Salivary gland surgery	N = 11180	N = 2652		
No	11169 (99.9%)	2650 (99.9%)		
Yes	11 (0.1%)	2 (0.1%)	0.712	0.98
Chronic ear illness	N = 2422	N = 562		
No	2288 (94.5%)	387 (68.9%)		
Yes	134 (5.5%)	175 (31.1%)	< 0.001	7.87
Chronic otitis externa	N = 2340	N = 452		
No	2333 (99.7%)	448 (99.1%)		
Yes	7 (0.3%)	4 (0.9%)	0.089	2.92
Chronic otitis media	N = 2340	N = 452		
No	2337 (99.9%)	449 (99.3%)		
Yes	3 (0.1%)	3 (0.7%)	0.048	2.25

Cholesteatom	N = 2340	N = 452		
No	2339 (100.0%)	452 (100.0%)		
Yes	1 (0.0%)	0 (0.0%)	0.999	0.0
Tympanostomy tube as child	N = 2313	N = 542		
No	2300 (99.4%)	538 (99.3%)		
Yes	13 (0.6%)	4 (0.7%)	0.623	1.33
Stroke	N = 11490	N = 2732		
No	11193 (97.4%)	2657 (97.3%)		
Yes	297 (2.6%)	75 (2.7%)	0.959	1.01
Seizure	N = 11507	N = 2739		
No	11272 (98.0%)	2698 (98.5%)		
Yes	235 (2.0%)	41 (1.5%)	0.07	0.73
Parkinson's	N = 11517	N = 2740		
No	11481 (99.7%)	2734 (99.8%)		
Yes	36 (0.3%)	6 (0.2%)	0.308	0.64
Multiple sclerosis	N = 11523	N = 2746		
No	11462 (99.5%)	2735 (99.6%)		
Yes	61 (0.5%)	11 (0.4%)	0.46	0.78
Disorder of carbohydrate metabolism	N = 2337	N = 556		
No	2077 (88.9%)	484 (87.1%)		
Yes	260 (11.1%)	72 (12.9%)	0.296	1.16
Cleft lip and palate	N = 2416	N = 580		
No	2414 (99.9%)	579 (99.8%)		
Yes	2 (0.1%)	1 (0.2%)	0.569	2.01

Table 9: Statistical analysis for the remaining medication using age- and sex- adjusted logistic regression.

Variable	No Tinnitus	Tinnitus	p-value	Odds Ratio
Diuretics	N = 11150	N = 2683		
No	10590 (95.0%)	2557 (95.3%)		
Yes	560 (5.0%)	126 (4.7%)	0.263	0.89
Aminoglycosides	N = 11150	N = 2683		
No	11149 (100.0%)	2683 (100.0%)		
Yes	1 (0.0%)	0 (0.0%)	-	-
Beta blocker	N = 11150	N = 2683		
No	11148 (100.0%)	2683 (100.0%)		
Yes	2 (0.0%)	0 (0.0%)	-	-
ACE inhibitor	N = 11150	N = 2683		
No	9986 (89.6%)	2406 (89.7%)		
Yes	1164 (10.4%)	277 (10.3%)	0.442	0.95

Table 10: Statistical analysis of subjective hearing problems with hearing aids using age- and sex-adjusted logistic regression. Answers are given on a scale from 1 (Always) to 5 (Never).

Variable	No Tinnitus	Tinnitus	p-value	Odds Ratio
Understand radio/Tv at room volume? With hearing aids	N = 45	N = 60		
1	24 (53.3%)	27 (45.0%)		
2	13 (28.9%)	28 (46.7%)		
3	6 (13.3%)	4 (6.7%)		
4	1 (2.2%)	1 (1.7%)		
5	1 (2.2%)	0 (0.0%)	0.479	0.83
Sudden loud traffic noise, know direction? With hearing aids	N = 40	N = 57		
1	19 (47.5%)	18 (31.6%)		
2	11 (27.5%)	21 (36.8%)		
3	7 (17.5%)	9 (15.8%)		
4	1 (2.5%)	4 (7.0%)		
5	2 (5.0%)	5 (8.8%)	0.242	1.26
Quite room, can hear phone or doorbell? With hearing aids	N = 45	N = 59		
1	33 (73.3%)	39 (66.1%)		
2	9 (20.0%)	15 (25.4%)		
3	1 (2.2%)	5 (8.5%)		
4	1 (2.2%)	0 (0.0%)		
5	1 (2.2%)	0 (0.0%)	0.938	0.98
Room with multiple persons, can understand conversations? With hearing aids	N = 45	N = 60		
1	16 (35.6%)	12 (20.0%)		
2	17 (37.8%)	32 (53.3%)		
3	10 (22.2%)	8 (13.3%)		
4	0 (0.0%)	8 (13.3%)		
5	2 (4.4%)	0 (0.0%)	0.316	1.26
Quite room, can hear door opening? With hearing aids	N = 44	N = 59		
1	29 (65.9%)	29 (49.2%)		
2	9 (20.5%)	24 (40.7%)		
3	4 (9.1%)	5 (8.5%)		
4	1 (2.3%)	1 (1.7%)		
5	1 (2.3%)	0 (0.0%)	0.602	1.15
Party, can easily have	N = 45	N = 58	0.198	1.29

conversations? With hearing aids				
1	13 (28.9%)	12 (20.7%)		
2	18 (40.0%)	18 (31.0%)		
3	8 (17.8%)	19 (32.8%)		
4	4 (8.9%)	5 (8.6%)		
5	2 (4.4%)	4 (6.9%)		
Walk in quite area, can easily have conversation? With hearing aids	N = 44	N = 58		
1	34 (77.3%)	41 (70.7%)		
2	8 (18.2%)	16 (27.6%)		
3	1 (2.3%)	1 (1.7%)		
4	0 (0.0%)	0 (0.0%)		
5	1 (2.3%)	0 (0.0%)	0.892	0.95
Car/Bus/Train, can easily have conversation? With hearing aids	N = 44	N = 58		
1	26 (59.1%)	28 (48.3%)		
2	14 (31.8%)	23 (39.7%)		
3	2 (4.5%)	6 (10.3%)		
4	0 (0.0%)	1 (1.7%)		
5	2 (4.5%)	0 (0.0%)	0.716	1.1
Can immediately tell where someone is speaking from? With hearing aids	N = 44	N = 59		
1	22 (50.0%)	22 (37.3%)		
2	16 (36.4%)	29 (49.2%)		
3	3 (6.8%)	3 (5.1%)		
4	1 (2.3%)	1 (1.7%)		
5	2 (4.5%)	4 (6.8%)	0.552	1.14
Quite room, can talk to someone without looking at the person? With hearing aids	N = 43	N = 59		
1	27 (62.8%)	31 (52.5%)		
2	10 (23.3%)	25 (42.4%)		
3	4 (9.3%)	2 (3.4%)		
4	0 (0.0%)	1 (1.7%)		
5	2 (4.7%)	0 (0.0%)	0.711	0.91
Room with keyboard noise, music, etc., can easily have conversation? With hearing aids	N = 44	N = 57		
1	19 (43.2%)	27 (47.4%)		
2	17 (38.6%)	23 (40.4%)		
3	5 (11.4%)	5 (8.8%)		
4	1 (2.3%)	1 (1.8%)		
5	2 (4.5%)	1 (1.8%)	0.477	0.85
Easily understand spoken words in echoing rooms? With hearing aids	N = 45<br>14 (31.1%)	N = 58<br>15 (25.9%)	0.33	1.23

1				
2	17 (37.8%)	26 (44.8%)		
3	12 (26.7%)	7 (12.1%)		
4	0 (0.0%)	9 (15.5%)		
5	2 (4.4%)	1 (1.7%)		

Table 11: Statistical analysis for the remaining noise sources in the living environment using age- and sex- adjusted logistic regression. Answers are given on a scale from 0 (Not at all) to 3 (Very much).

Variable	No Tinnitus	Tinnitus	p-value	Odds Ratio
Feel disturbed by rail traffic noise	N = 11139	N = 2662		
0	10046 (90.2%)	2390 (89.8%)		
1	920 (8.3%)	232 (8.7%)		
2	135 (1.2%)	32 (1.2%)		
3	38 (0.3%)	8 (0.3%)	0.653	1.03
Feel disturbed by port noise	N = 11089	N = 2662		
0	10701 (96.5%)	2551 (95.8%)		
1	345 (3.1%)	103 (3.9%)		
2	39 (0.4%)	7 (0.3%)		
3	4 (0.0%)	1 (0.0%)	0.181	1.14
Feel disturbed by animal noise	N = 11172	N = 2673		
0	10160 (90.9%)	2389 (89.4%)		
1	931 (8.3%)	257 (9.6%)		
2	67 (0.6%)	22 (0.8%)		
3	14 (0.1%)	5 (0.2%)	0.006	1.18

Table 12: Statistical analysis of noise at work using age- and sex- adjusted logistic regression. Answers are given on a scale from 1 (Always) to 5 (Never).

Variable	No Tinnitus	Tinnitus	p-value	Odds Ratio
How often changing acustics at work?	N = 375	N = 228		
1	39 (10.4%)	26 (11.4%)		
2	138 (36.8%)	72 (31.6%)		
3	87 (23.2%)	68 (29.8%)		
4	88 (23.5%)	51 (22.4%)		
5	23 (6.1%)	11 (4.8%)	0.993	1.0

How often client/employee conversations?	N = 375	N = 227		
1	63 (16.8%)	35 (15.4%)		
2	196 (52.3%)	112 (49.3%)		
3	61 (16.3%)	42 (18.5%)		
4	27 (7.2%)	19 (8.4%)		
5	28 (7.5%)	19 (8.4%)	0.343	1.08
How often professional development opportunities?	N = 372	N = 226		
1	6 (1.6%)	2 (0.9%)		
2	58 (15.6%)	29 (12.8%)		
3	135 (36.3%)	96 (42.5%)		
4	125 (33.6%)	72 (31.9%)		
5	48 (12.9%)	27 (11.9%)	0.95	1.01
How often specific hygiene requirements?	N = 371	N = 227		
1	46 (12.4%)	24 (10.6%)		
2	56 (15.1%)	24 (10.6%)		
3	42 (11.3%)	21 (9.3%)		
4	88 (23.7%)	51 (22.5%)		
5	139 (37.5%)	107 (47.1%)	0.028	1.14
How often phone calls for work?	N = 377	N = 229		
1	55 (14.6%)	34 (14.8%)		
2	210 (55.7%)	130 (56.8%)		
3	61 (16.2%)	42 (18.3%)		
4	38 (10.1%)	16 (7.0%)		
5	13 (3.4%)	7 (3.1%)	0.483	0.94
How often have to understand speech in noisy environments?	N = 375	N = 225		
1	35 (9.3%)	29 (12.9%)		
2	130 (34.7%)	81 (36.0%)		
3	118 (31.5%)	71 (31.6%)		
4	70 (18.7%)	36 (16.0%)		
5	22 (5.9%)	8 (3.6%)	0.07	0.86
How often meetings with multiple colleagues?	N = 377	N = 228		
1	24 (6.4%)	10 (4.4%)		
2	168 (44.6%)	122 (53.5%)		
3	115 (30.5%)	64 (28.1%)		
4	50 (13.3%)	27 (11.8%)		
5	20 (5.3%)	5 (2.2%)	0.094	0.86
How often need to pay attention	N = 362	N = 225		
to warning signals/machine noise?	20 (5.5%)	9 (4.0%)		
1	25 (6.9%)	18 (8.0%)		
2	47 (13.0%)	22 (9.8%)		
3	99 (27.3%)	55 (24.4%)	0.164	1.11

4				
5	171 (47.2%)	121 (53.8%)		
How often NOT need to talk to others at work?	N = 370	N = 225		
1	0 (0.0%)	2 (0.9%)		
2	52 (14.1%)	40 (17.8%)		
3	84 (22.7%)	50 (22.2%)		
4	163 (44.1%)	97 (43.1%)		
5	71 (19.2%)	36 (16.0%)	0.118	0.87
How often other communication/hearing requirements at work?	N = 221	N = 140		
1	46 (20.8%)	28 (20.0%)		
2	90 (40.7%)	63 (45.0%)		
3	35 (15.8%)	26 (18.6%)		
4	9 (4.1%)	7 (5.0%)		
5	41 (18.6%)	16 (11.4%)	0.23	0.9

Unadjusted logistic regression

Table 13: Statistical analysis of demographic and lifestyle factors using unadjusted logistic regression.

Variable	No Tinnitus	Tinnitus	p-value	Odds Ratio
Age	N = 11590	N = 2765		
60.6 [60.5, 60.7]	60.5 [60.3, 60.7]	61.0 [60.7, 61.3]	0.006	1.01
Sex	N = 11590	N = 2765		
Male	5693 (49.1%)	1463 (52.9%)		
Female	5897 (50.9%)	1302 (47.1%)	< 0.001	0.86
Are you smoking?	N = 11533	N = 2752		
Yes	2410 (20.9%)	519 (18.9%)	0.017	0.88
No (min. 6 Month)	4646 (40.3%)	1214 (44.1%)	< 0.001	1.17
No (never)	4477 (38.8%)	1019 (37.0%)	0.083	0.93
Martial Status	N = 11417	N = 2721		
Married, living together	7223 (63.3%)	1725 (63.4%)	0.899	1.01
Married, living separately	308 (2.7%)	70 (2.6%)	0.716	0.95
Single	1752 (15.3%)	401 (14.7%)	0.427	0.95
Divorced	1504 (13.2%)	377 (13.9%)	0.347	1.06
Widowed	630 (5.5%)	148 (5.4%)	0.871	0.98
Commited Relationship	N = 6962	N = 1670	0.653	1.03
No	2228 (32.0%)	544 (32.6%)	0.391	0.95
Yes, permanently living together	3783 (54.3%)	888 (53.2%)	0.466	0.93

Yes, partially living together	524 (7.5%)	117 (7.0%)		
Yes, living separately	427 (6.1%)	121 (7.2%)	0.095	1.2
Employment Status	N = 10834	N = 2579		
Full-time	4753 (43.9%)	1136 (44.0%)	0.871	1.01
Part-time	1732 (16.0%)	328 (12.7%)	< 0.001	0.77
Partial retirement	131 (1.2%)	35 (1.4%)	0.542	1.12
Minijob	387 (3.6%)	98 (3.8%)	0.578	1.07
Irregularly employed	117 (1.1%)	34 (1.3%)	0.303	1.22
Not employed	3714 (34.3%)	948 (36.8%)	0.018	1.11
	N = 6576	N = 1511		
Since when employed? (year) 1993.8 [1993.5, 1994.1]	1993.9 [1993.6, 1994.2]	1993.3 [1992.6, 1993.9]	0.097	1.0
Working hours	N = 5968	N = 1356		
34.9 [34.6, 35.2]	34.8 [34.5, 35.2]	35.2 [34.5, 35.8]	0.368	1.0
Occupational status if unemployed	N = 3624	N = 930		
Student	3 (0.1%)	4 (0.4%)	0.031	5.21
Retiree	3056 (84.3%)	779 (83.8%)	0.674	0.96
Unemployed	168 (4.6%)	40 (4.3%)	0.663	0.92
Permanently unable to work	66 (1.8%)	31 (3.3%)	0.005	1.86
Homemaker	220 (6.1%)	41 (4.4%)	0.053	0.71
Other	111 (3.1%)	35 (3.8%)	0.28	1.24
Ever worked full/part-time?	N = 2438	N = 664		
No	65 (2.7%)	20 (3.0%)		
Yes	2373 (97.3%)	644 (97.0%)	0.629	0.88
Ever been unemployed?	N = 11033	N = 2645		
No	5626 (51.0%)	1294 (48.9%)		
Yes	5407 (49.0%)	1351 (51.1%)	0.056	1.09
How many times unemployed?	N = 4466	N = 1106		
1.8 [1.7, 1.8]	1.8 [1.7, 1.9]	1.8 [1.7, 1.8]	0.557	0.99
How long unemployed? (months)	N = 4249	N = 1049		
17.8 [17.0, 18.7]	17.9 [16.9, 18.9]	17.5 [15.6, 19.4]	0.693	1.0
Currently unemployed?	N = 5165	N = 1302		
No	4899 (94.8%)	1225 (94.1%)		
Yes	266 (5.2%)	77 (5.9%)	0.272	1.16
Occupational group	N = 10590	N = 2540		
Academic	656 (6.2%)	120 (4.7%)	0.005	0.75
Self-employed	1241 (11.7%)	268 (10.6%)	0.098	0.89
Civil servant	874 (8.3%)	275 (10.8%)	< 0.001	1.35
Emloyee	7017 (66.3%)	1698 (66.9%)	0.572	1.03
Laborer	802 (7.6%)	179 (7.0%)	0.365	0.93
Ever worked night-shifts?	N = 10708	N = 2569		
No	8837 (82.5%)	2069 (80.5%)		
Yes	1871 (17.5%)	500 (19.5%)	0.018	1.14
Net income (1=<500€ -17=8000€+)	N = 9353	N = 2234		
1	485 (5.2%)	95 (4.3%)	0.04	1.01

2	310 (3.3%)	50 (2.2%)		
3	550 (5.9%)	113 (5.1%)		
4	620 (6.6%)	157 (7.0%)		
5	726 (7.8%)	173 (7.7%)		
6	697 (7.5%)	172 (7.7%)		
7	797 (8.5%)	191 (8.5%)		
8	795 (8.5%)	197 (8.8%)		
9	714 (7.6%)	161 (7.2%)		
10	966 (10.3%)	245 (11.0%)		
11	752 (8.0%)	189 (8.5%)		
12	450 (4.8%)	134 (6.0%)		
13	359 (3.8%)	98 (4.4%)		
14	269 (2.9%)	69 (3.1%)		
15	310 (3.3%)	71 (3.2%)		
16	251 (2.7%)	54 (2.4%)		
17	302 (3.2%)	65 (2.9%)		
Household income (1=<500€ -17=8000€+)	N = 8861	N = 2117		
1	64 (0.7%)	9 (0.4%)		
2	63 (0.7%)	18 (0.9%)		
3	181 (2.0%)	42 (2.0%)		
4	245 (2.8%)	57 (2.7%)		
5	320 (3.6%)	65 (3.1%)		
6	307 (3.5%)	84 (4.0%)		
7	422 (4.8%)	115 (5.4%)		
8	481 (5.4%)	132 (6.2%)		
9	536 (6.0%)	122 (5.8%)		
10	905 (10.2%)	193 (9.1%)		
11	948 (10.7%)	239 (11.3%)		
12	778 (8.8%)	184 (8.7%)		
13	679 (7.7%)	164 (7.7%)		
14	627 (7.1%)	163 (7.7%)		
15	848 (9.6%)	207 (9.8%)		
16	758 (8.6%)	183 (8.6%)		
17	699 (7.9%)	140 (6.6%)	0.638	1.0
Housing type	N = 11478	N = 2733		
Detached single-family house	2391 (20.8%)	536 (19.6%)	0.157	0.93
Semi-detached house	1067 (9.3%)	260 (9.5%)	0.726	1.03
Terraced house	1343 (11.7%)	341 (12.5%)	0.259	1.08
Multi-family house	5964 (52.0%)	1439 (52.7%)	0.515	1.03
high-rise building	481 (4.2%)	100 (3.7%)	0.208	0.87
Other	232 (2.0%)	57 (2.1%)	0.83	1.03
Rent/ownership	N = 11461	N = 2734		
Rent	5565 (48.6%)	1330 (48.6%)	0.932	1.0
Ownership	5811 (50.7%)	1385 (50.7%)	0.967	1.0
Other	85 (0.7%)	19 (0.7%)	0.797	0.94

Table 14: Statistical analysis of comorbidities using unadjusted logistic regression.

Variable	No Tinnitus	Tinnitus	p-value	Odds Ratio
Heart Attack	N = 11502	N = 2736		
No	11186 (97.3%)	2654 (97.0%)		
Yes	316 (2.7%)	82 (3.0%)	0.476	1.09
Coronary artery disease	N = 11251	N = 2660		
No	10760 (95.6%)	2519 (94.7%)		
Yes	491 (4.4%)	141 (5.3%)	0.037	1.23
Angia pectoris	N = 10610	N = 2529		
No	10235 (96.5%)	2421 (95.7%)		
Yes	375 (3.5%)	108 (4.3%)	0.078	1.22
Claudication	N = 11202	N = 2634		
No	10838 (96.8%)	2541 (96.5%)		
Yes	364 (3.2%)	93 (3.5%)	0.467	1.09
Carotid stenosis	N = 11169	N = 2636		
No	10736 (96.1%)	2505 (95.0%)		
Yes	433 (3.9%)	131 (5.0%)	0.011	1.3
Had surgery in ENT region?	N = 11338	N = 2691		
No	8035 (70.9%)	1702 (63.2%)		
Yes	3303 (29.1%)	989 (36.8%)	< 0.001	1.41
Nasal conchae surgery?	N = 11180	N = 2652		
No	11107 (99.3%)	2618 (98.7%)		
Yes	73 (0.7%)	34 (1.3%)	0.001	1.98
Nasal septum surgery?	N = 11180	N = 2652		
No	10977 (98.2%)	2562 (96.6%)		
Yes	203 (1.8%)	90 (3.4%)	< 0.001	1.38
Paranasal sinus surgery?	N = 11180	N = 2652		
No	11030 (98.7%)	2592 (97.7%)		
Yes	150 (1.3%)	60 (2.3%)	< 0.001	1.19
External nose surgery?	N = 11180	N = 2652		
No	11168 (99.9%)	2645 (99.7%)		
Yes	12 (0.1%)	7 (0.3%)	0.058	1.25
Outer ear surgery?	N = 11180	N = 2652		
No	11156 (99.8%)	2644 (99.7%)		
Yes	24 (0.2%)	8 (0.3%)	0.404	1.07
Middle ear surgery?	N = 11180	N = 2652		
No	11154 (99.8%)	2636 (99.4%)		
Yes	26 (0.2%)	16 (0.6%)	0.003	1.17
Inner ear surgery?	N = 11180	N = 2652		
No	11172 (99.9%)	2642 (99.6%)		
Yes	8 (0.1%)	10 (0.4%)	< 0.001	1.27
Pharynx surgery via mouth?	N = 11180	N = 2652	0.107	1.03

No	10995 (98.3%)	2596 (97.9%)		
Yes	185 (1.7%)	56 (2.1%)		
Pharynx surgery external?	N = 11180	N = 2652		
No	11173 (99.9%)	2649 (99.9%)		
Yes	7 (0.1%)	3 (0.1%)	0.391	1.07
Larynx surgery via mouth?	N = 11180	N = 2652		
No	11175 (100.0%)	2648 (99.8%)		
Yes	5 (0.0%)	4 (0.2%)	0.07	1.13
Larynx surgery external?	N = 11180	N = 2652		
No	11178 (100.0%)	2650 (99.9%)		
Yes	2 (0.0%)	2 (0.1%)	0.15	1.14
Neck soft tissue surgery?	N = 11180	N = 2652		
No	11158 (99.8%)	2648 (99.8%)		
Yes	22 (0.2%)	4 (0.2%)	0.624	0.98
Salivary gland surgery?	N = 11180	N = 2652		
No	11169 (99.9%)	2650 (99.9%)		
Yes	11 (0.1%)	2 (0.1%)	0.729	0.98
Chronic ear illness	N = 2422	N = 562		
No	2288 (94.5%)	387 (68.9%)		
Yes	134 (5.5%)	175 (31.1%)	< 0.001	7.72
Chronic otitis externa?	N = 2340	N = 452		
No	2333 (99.7%)	448 (99.1%)		
Yes	7 (0.3%)	4 (0.9%)	0.083	2.98
Chronic otitis media?	N = 2340	N = 452		
No	2337 (99.9%)	449 (99.3%)		
Yes	3 (0.1%)	3 (0.7%)	0.044	2.28
Cholesteatom?	N = 2340	N = 452		
No	2339 (100.0%)	452 (100.0%)		
Yes	1 (0.0%)	0 (0.0%)	0.999	0.0
Conductive hearing loss?	N = 2340	N = 452		
No	2321 (99.2%)	419 (92.7%)		
Yes	19 (0.8%)	33 (7.3%)	< 0.001	1.76
Sensorineural hearing loss?	N = 2340	N = 452		
No	2316 (99.0%)	413 (91.4%)		
Yes	24 (1.0%)	39 (8.6%)	< 0.001	1.56
Hearing aid	N = 2444	N = 585		
No	2399 (98.2%)	526 (89.9%)		
Yes	45 (1.8%)	59 (10.1%)	< 0.001	5.98
Frequent Otitis media as child	N = 2221	N = 518		
No	1954 (88.0%)	432 (83.4%)		
Yes	267 (12.0%)	86 (16.6%)	0.005	1.46
Tympanostomy tube as child	N = 2313	N = 542		
No	2300 (99.4%)	538 (99.3%)		
Yes	13 (0.6%)	4 (0.7%)	0.633	1.32
sleep apnea	N = 11212	N = 2677	< 0.001	1.4

No	10639 (94.9%)	2489 (93.0%)		
Yes	573 (5.1%)	188 (7.0%)		
Stroke	N = 11490	N = 2732		
No	11193 (97.4%)	2657 (97.3%)		
Yes	297 (2.6%)	75 (2.7%)	0.637	1.06
Memory deterioration	N = 10240	N = 2430		
No	5625 (54.9%)	1136 (46.7%)		
Yes	4615 (45.1%)	1294 (53.3%)	< 0.001	1.39
Seizure	N = 11507	N = 2739		
No	11272 (98.0%)	2698 (98.5%)		
Yes	235 (2.0%)	41 (1.5%)	0.064	0.73
Parkinson's	N = 11517	N = 2740		
No	11481 (99.7%)	2734 (99.8%)		
Yes	36 (0.3%)	6 (0.2%)	0.419	0.7
Multiple sclerosis	N = 11523	N = 2746		
No	11462 (99.5%)	2735 (99.6%)		
Yes	61 (0.5%)	11 (0.4%)	0.394	0.76
Migraine	N = 11419	N = 2711		
No	10156 (88.9%)	2282 (84.2%)		
Yes	1263 (11.1%)	429 (15.8%)	< 0.001	1.51
Disorder of carbohydrate metabolism	N = 2337	N = 556		
No	2077 (88.9%)	484 (87.1%)		
Yes	260 (11.1%)	72 (12.9%)	0.226	1.19
Shingles	N = 11380	N = 2725		
No	9675 (85.0%)	2244 (82.3%)		
Yes	1705 (15.0%)	481 (17.7%)	< 0.001	1.22
Hypertension	N = 11442	N = 2728		
No	7482 (65.4%)	1721 (63.1%)		
Yes	3960 (34.6%)	1007 (36.9%)	0.023	1.11
Diabetes mellitus	N = 10783	N = 2599		
No	9802 (90.9%)	2397 (92.2%)		
Yes	981 (9.1%)	202 (7.8%)	0.033	0.84
Cleft lip and palate	N = 2416	N = 580		
No	2414 (99.9%)	579 (99.8%)		
Yes	2 (0.1%)	1 (0.2%)	0.549	2.08

Table 15: Statistical analysis of medication using unadjusted logistic regression.

Variable	No Tinnitus	Tinnitus	p-value	Odds Ratio
Acetylsalicylic acid	N = 11150	N = 2683		
No	10098 (90.6%)	2391 (89.1%)	0.023	1.17

Yes	1052 (9.4%)	292 (10.9%)		
Diuretics	N = 11150	N = 2683		
No	10590 (95.0%)	2557 (95.3%)		
Yes	560 (5.0%)	126 (4.7%)	0.485	0.93
Antidepressants	N = 11150	N = 2683		
No	10574 (94.8%)	2464 (91.8%)		
Yes	576 (5.2%)	219 (8.2%)	< 0.001	1.63
Aminoglycosides	N = 11150	N = 2683		
No	11149 (100.0%)	2683 (100.0%)		
Yes	1 (0.0%)	0 (0.0%)	-	-
Beta blocker	N = 11150	N = 2683		
No	11148 (100.0%)	2683 (100.0%)		
Yes	2 (0.0%)	0 (0.0%)	-	-
ACE inhibitor	N = 11150	N = 2683		
No	9986 (89.6%)	2406 (89.7%)		
Yes	1164 (10.4%)	277 (10.3%)	0.861	0.99
Medications (general)	N = 11150	N = 2683		
No	3117 (28.0%)	624 (23.3%)		
Yes	8033 (72.0%)	2059 (76.7%)	< 0.001	1.28

Table 16: Statistical analysis of subjective hearing problems using unadjusted logistic regression.. Answers are given on a scale from 1 (Always) to 5 (Never).

Variable	No Tinnitus	Tinnitus	p-value	Odds Ratio
Understand radio/Tv at room volume?	N = 390	N = 235		
1	244 (62.6%)	85 (36.2%)		
2	93 (23.8%)	70 (29.8%)		
3	35 (9.0%)	42 (17.9%)		
4	10 (2.6%)	26 (11.1%)		
5	8 (2.1%)	12 (5.1%)	< 0.001	1.74
Sudden loud traffic noise, know direction?	N = 385	N = 233		
1	240 (62.3%)	99 (42.5%)		
2	111 (28.8%)	74 (31.8%)		
3	22 (5.7%)	28 (12.0%)		
4	6 (1.6%)	19 (8.2%)		
5	6 (1.6%)	13 (5.6%)	< 0.001	1.7
Quite room, can hear phone or	N = 391	N = 236		
doorbell?	341 (87.2%)	182 (77.1%)		
1	34 (8.7%)	29 (12.3%)		
2	8 (2.0%)	10 (4.2%)		
3	6 (1.5%)	11 (4.7%)		
4	2 (0.5%)	4 (1.7%)	< 0.001	1.5

5				
Room with multiple persons, can understand conversations?	N = 391	N = 235		
1	182 (46.5%)	46 (19.6%)		
2	127 (32.5%)	83 (35.3%)		
3	54 (13.8%)	51 (21.7%)		
4	19 (4.9%)	43 (18.3%)		
5	9 (2.3%)	12 (5.1%)	< 0.001	1.81
Quite room, can hear door opening?	N = 390	N = 236		
1	308 (79.0%)	144 (61.0%)		
2	63 (16.2%)	52 (22.0%)		
3	10 (2.6%)	24 (10.2%)		
4	6 (1.5%)	12 (5.1%)		
5	3 (0.8%)	4 (1.7%)	< 0.001	1.71
Party, can easily have conversations?	N = 388	N = 232		
1	120 (30.9%)	31 (13.4%)		
2	150 (38.7%)	73 (31.5%)		
3	70 (18.0%)	48 (20.7%)		
4	34 (8.8%)	63 (27.2%)		
5	14 (3.6%)	17 (7.3%)	< 0.001	1.69
Walk in quite area, can easily have conversation?	N = 389	N = 234		
1	324 (83.3%)	157 (67.1%)		
2	50 (12.9%)	53 (22.6%)		
3	11 (2.8%)	15 (6.4%)		
4	2 (0.5%)	5 (2.1%)		
5	2 (0.5%)	4 (1.7%)	< 0.001	1.73
Car/Bus/Train, can easily have conversation?	N = 390	N = 235		
1	252 (64.6%)	98 (41.7%)		
2	105 (26.9%)	92 (39.1%)		
3	24 (6.2%)	22 (9.4%)		
4	6 (1.5%)	17 (7.2%)		
5	3 (0.8%)	6 (2.6%)	< 0.001	1.75
Can immediately tell where someone is speaking from?	N = 388	N = 236		
1	271 (69.8%)	116 (49.2%)		
2	100 (25.8%)	74 (31.4%)		
3	8 (2.1%)	27 (11.4%)		
4	5 (1.3%)	12 (5.1%)		
5	4 (1.0%)	7 (3.0%)	< 0.001	1.83
Quite room, can talk to	N = 383	N = 233		
someone without looking at the	297 (77.5%)	136 (58.4%)		
person?	63 (16.4%)	61 (26.2%)	< 0.001	1.65

1				
2				
3	15 (3.9%)	21 (9.0%)		
4	4 (1.0%)	10 (4.3%)		
5	4 (1.0%)	5 (2.1%)		
Room with keyboard noise, music, etc., can easily have conversation?	N = 383	N = 233		
1	222 (58.0%)	91 (39.1%)		
2	115 (30.0%)	82 (35.2%)		
3	35 (9.1%)	41 (17.6%)		
4	9 (2.3%)	15 (6.4%)		
5	2 (0.5%)	4 (1.7%)	< 0.001	1.63
Easily understand spoken words in echoing rooms?	N = 388	N = 231		
1	158 (40.7%)	55 (23.8%)		
2	153 (39.4%)	89 (38.5%)		
3	51 (13.1%)	47 (20.3%)		
4	19 (4.9%)	28 (12.1%)		
5	7 (1.8%)	12 (5.2%)	< 0.001	1.57

Table 17: Statistical analysis of subjective hearing problems with hearing aids using unadjusted logistic regression. Answers are given on a scale from 1 (Always) to 5 (Never).

Variable	No Tinnitus	Tinnitus	p-value	Odds Ratio
Understand radio/Tv at room volume? With hearing aids	N = 45	N = 60		
1	24 (53.3%)	27 (45.0%)		
2	13 (28.9%)	28 (46.7%)		
3	6 (13.3%)	4 (6.7%)		
4	1 (2.2%)	1 (1.7%)		
5	1 (2.2%)	0 (0.0%)	0.698	0.91
Sudden loud traffic noise, know direction? With hearing aids	N = 40	N = 57		
1	19 (47.5%)	18 (31.6%)		
2	11 (27.5%)	21 (36.8%)		
3	7 (17.5%)	9 (15.8%)		
4	1 (2.5%)	4 (7.0%)		
5	2 (5.0%)	5 (8.8%)	0.161	1.3
Quite room, can hear phone or doorbell? With hearing aids	N = 45	N = 59		
1	33 (73.3%)	39 (66.1%)		
2	9 (20.0%)	15 (25.4%)		
3	1 (2.2%)	5 (8.5%)		
4	1 (2.2%)	0 (0.0%)		
5	1 (2.2%)	0 (0.0%)	0.869	1.05

Room with multiple persons, can understand conversations? With hearing aids	N = 45	N = 60		
1	16 (35.6%)	12 (20.0%)		
2	17 (37.8%)	32 (53.3%)		
3	10 (22.2%)	8 (13.3%)		
4	0 (0.0%)	8 (13.3%)		
5	2 (4.4%)	0 (0.0%)	0.288	1.26
Quite room, can hear door opening? With hearing aids	N = 44	N = 59		
1	29 (65.9%)	29 (49.2%)		
2	9 (20.5%)	24 (40.7%)		
3	4 (9.1%)	5 (8.5%)		
4	1 (2.3%)	1 (1.7%)		
5	1 (2.3%)	0 (0.0%)	0.612	1.14
Party, can easily have conversations? With hearing aids	N = 45	N = 58		
1	13 (28.9%)	12 (20.7%)		
2	18 (40.0%)	18 (31.0%)		
3	8 (17.8%)	19 (32.8%)		
4	4 (8.9%)	5 (8.6%)		
5	2 (4.4%)	4 (6.9%)	0.179	1.28
Walk in quite area, can easily have conversation? With hearing aids	N = 44	N = 58		
1	34 (77.3%)	41 (70.7%)		
2	8 (18.2%)	16 (27.6%)		
3	1 (2.3%)	1 (1.7%)		
4	0 (0.0%)	0 (0.0%)		
5	1 (2.3%)	0 (0.0%)	0.949	0.98
Car/Bus/Train, can easily have conversation? With hearing aids	N = 44	N = 58		
1	26 (59.1%)	28 (48.3%)		
2	14 (31.8%)	23 (39.7%)		
3	2 (4.5%)	6 (10.3%)		
4	0 (0.0%)	1 (1.7%)		
5	2 (4.5%)	0 (0.0%)	0.698	1.1
Can immediately tell where someone is speaking from? With hearing aids	N = 44	N = 59		
1	22 (50.0%)	22 (37.3%)		
2	16 (36.4%)	29 (49.2%)		
3	3 (6.8%)	3 (5.1%)		
4	1 (2.3%)	1 (1.7%)		
5	2 (4.5%)	4 (6.8%)	0.424	1.17

Quite room, can talk to someone without looking at the person? With hearing aids	N = 43	N = 59		
1	27 (62.8%)	31 (52.5%)		
2	10 (23.3%)	25 (42.4%)		
3	4 (9.3%)	2 (3.4%)		
4	0 (0.0%)	1 (1.7%)		
5	2 (4.7%)	0 (0.0%)	0.702	0.91
Room with keyboard noise, music, etc., can easily have conversation? With hearing aids	N = 44	N = 57		
1	19 (43.2%)	27 (47.4%)		
2	17 (38.6%)	23 (40.4%)		
3	5 (11.4%)	5 (8.8%)		
4	1 (2.3%)	1 (1.8%)		
5	2 (4.5%)	1 (1.8%)	0.385	0.83
Easily understand spoken words in echoing rooms? With hearing aids	N = 45	N = 58		
1	14 (31.1%)	15 (25.9%)		
2	17 (37.8%)	26 (44.8%)		
3	12 (26.7%)	7 (12.1%)		
4	0 (0.0%)	9 (15.5%)		
5	2 (4.4%)	1 (1.7%)	0.507	1.14

Table 18: Statistical analysis of the social effects of hearing problems using unadjusted logistic regression.. Answers are given on a scale from 1 (Never) to 11 (Always).

Variable	No Tinnitus	Tinnitus	p-value	Odds Ratio
Hearing loss affects interactions	N = 388	N = 237		
with others?	191 (49.2%)	44 (18.6%)		
1	35 (9.0%)	23 (9.7%)		
2	45 (11.6%)	30 (12.7%)		
3	4 (1.0%)	8 (3.4%)		
4	25 (6.4%)	11 (4.6%)		
5	51 (13.1%)	55 (23.2%)		
6	14 (3.6%)	17 (7.2%)		
7	3 (0.8%)	12 (5.1%)		
8	13 (3.4%)	24 (10.1%)		
9	4 (1.0%)	8 (3.4%)	< 0.001	1.28

10				
11	3 (0.8%)	5 (2.1%)		
Avoid meetings because of difficulties following conversations?	N = 388	N = 236		
1	308 (79.4%)	139 (58.9%)		
2	19 (4.9%)	17 (7.2%)		
3	26 (6.7%)	23 (9.7%)		
4	4 (1.0%)	6 (2.5%)		
5	6 (1.5%)	3 (1.3%)		
6	14 (3.6%)	30 (12.7%)		
7	4 (1.0%)	7 (3.0%)		
8	2 (0.5%)	1 (0.4%)		
9	3 (0.8%)	8 (3.4%)		
10	2 (0.5%)	2 (0.8%)		
11	0 (0.0%)	0 (0.0%)	< 0.001	1.26
Feel like people ignore you because of hearing problems?	N = 388	N = 237		
1	349 (89.9%)	182 (76.8%)		
2	11 (2.8%)	14 (5.9%)		
3	15 (3.9%)	21 (8.9%)		
4	2 (0.5%)	5 (2.1%)		
5	2 (0.5%)	1 (0.4%)		
6	5 (1.3%)	12 (5.1%)		
7	1 (0.3%)	1 (0.4%)		
8	0 (0.0%)	0 (0.0%)		
9	3 (0.8%)	1 (0.4%)		
10	0 (0.0%)	0 (0.0%)		
11	0 (0.0%)	0 (0.0%)	0.001	1.26
Feel like others find it difficult to hold conversation with you?	N = 388	N = 237		
1	276 (71.1%)	118 (49.8%)		
2	38 (9.8%)	28 (11.8%)		
3	37 (9.5%)	40 (16.9%)		
4	4 (1.0%)	8 (3.4%)		
5	9 (2.3%)	9 (3.8%)		
6	15 (3.9%)	25 (10.5%)		
7	4 (1.0%)	3 (1.3%)		
8	1 (0.3%)	2 (0.8%)		
9	3 (0.8%)	4 (1.7%)		
10	0 (0.0%)	0 (0.0%)		
11	1 (0.3%)	0 (0.0%)	< 0.001	1.25
Feel excluded because of	N = 388	N = 237		
hearing problems?	299 (77.1%)	133 (56.1%)		
1	21 (5.4%)	24 (10.1%)		
2	28 (7.2%)	34 (14.3%)	< 0.001	1.24

3				
4	6 (1.5%)	6 (2.5%)		
5	8 (2.1%)	5 (2.1%)		
6	17 (4.4%)	20 (8.4%)		
7	7 (1.8%)	5 (2.1%)		
8	0 (0.0%)	4 (1.7%)		
9	2 (0.5%)	5 (2.1%)		
10	0 (0.0%)	1 (0.4%)		
11	0 (0.0%)	0 (0.0%)		
Hesitate to meet new people because of hearing problems?	N = 386	N = 237		
1	356 (92.2%)	189 (79.7%)		
2	5 (1.3%)	8 (3.4%)		
3	11 (2.8%)	13 (5.5%)		
4	5 (1.3%)	7 (3.0%)		
5	2 (0.5%)	4 (1.7%)		
6	4 (1.0%)	10 (4.2%)		
7	1 (0.3%)	1 (0.4%)		
8	1 (0.3%)	1 (0.4%)		
9	1 (0.3%)	4 (1.7%)		
10	0 (0.0%)	0 (0.0%)		
11	0 (0.0%)	0 (0.0%)	< 0.001	1.32
Avoid group conversations, because of fear to give inappropriate answers?	N = 387	N = 237		
1	307 (79.3%)	141 (59.5%)		
2	22 (5.7%)	18 (7.6%)		
3	27 (7.0%)	28 (11.8%)		
4	3 (0.8%)	5 (2.1%)		
5	4 (1.0%)	5 (2.1%)		
6	15 (3.9%)	25 (10.5%)		
7	5 (1.3%)	2 (0.8%)		
8	0 (0.0%)	5 (2.1%)		
9	3 (0.8%)	5 (2.1%)		
10	0 (0.0%)	2 (0.8%)		
11	1 (0.3%)	1 (0.4%)	< 0.001	1.26
Hearing problems affect self-	N = 387	N = 236		
confidence?	322 (83.2%)	150 (63.6%)		
1	20 (5.2%)	21 (8.9%)		
2	16 (4.1%)	25 (10.6%)		
3	9 (2.3%)	2 (0.8%)		
4	5 (1.3%)	8 (3.4%)		
5	9 (2.3%)	19 (8.1%)		
6	2 (0.5%)	3 (1.3%)		
7	1 (0.3%)	3 (1.3%)		
8	2 (0.5%)	4 (1.7%)	< 0.001	1.28

9				
10	0 (0.0%)	1 (0.4%)		
11	1 (0.3%)	0 (0.0%)		
Feel inferior because of hearing problems?	N = 387	N = 237		
1	344 (88.9%)	174 (73.4%)		
2	12 (3.1%)	20 (8.4%)		
3	12 (3.1%)	23 (9.7%)		
4	4 (1.0%)	5 (2.1%)		
5	7 (1.8%)	4 (1.7%)		
6	6 (1.6%)	7 (3.0%)		
7	0 (0.0%)	0 (0.0%)		
8	1 (0.3%)	2 (0.8%)		
9	1 (0.3%)	1 (0.4%)		
10	0 (0.0%)	1 (0.4%)		
11	0 (0.0%)	0 (0.0%)	< 0.001	1.27
Feel sad or frustrated when not able participate in conversation because of hearing problems?	N = 387	N = 237		
1	284 (73.4%)	125 (52.7%)		
2	26 (6.7%)	23 (9.7%)		
3	23 (5.9%)	25 (10.5%)		
4	13 (3.4%)	6 (2.5%)		
5	12 (3.1%)	8 (3.4%)		
6	18 (4.7%)	33 (13.9%)		
7	6 (1.6%)	6 (2.5%)		
8	2 (0.5%)	2 (0.8%)		
9	2 (0.5%)	4 (1.7%)		
10	1 (0.3%)	3 (1.3%)		
11	0 (0.0%)	2 (0.8%)	< 0.001	1.24

Table 19: Statistical analysis of noise disturbance in the living environment using unadjusted logistic regression. Answers are given on a scale from 0 (Not at all) to 3 (Very much).

Variable	No Tinnitus	Tinnitus	p-value	Odds

				**Ratio**
Feel disturbed by noise at home				
- weekdays, daytime	N = 11425	N = 2722		
0	6794 (59.5%)	1501 (55.1%)		
1	3853 (33.7%)	988 (36.3%)		
2	626 (5.5%)	177 (6.5%)		
3	152 (1.3%)	56 (2.1%)	< 0.001	1.16
Feel disturbed by noise at home				
- weekends, daytime	N = 11399	N = 2717		
0	7008 (61.5%)	1554 (57.2%)		
1	3735 (32.8%)	958 (35.3%)		
2	542 (4.8%)	161 (5.9%)		
3	114 (1.0%)	44 (1.6%)	< 0.001	1.17
Feel disturbed by noise at home				
- nighttime	N = 11399	N = 2712		
0	8553 (75.0%)	1968 (72.6%)		
1	2417 (21.2%)	618 (22.8%)		
2	321 (2.8%)	94 (3.5%)		
3	108 (0.9%)	32 (1.2%)	0.004	1.11
Feel disturbed by road traffic				
noise	N = 11280	N = 2696		
0	6874 (60.9%)	1560 (57.9%)		
1	3469 (30.8%)	874 (32.4%)		
2	661 (5.9%)	186 (6.9%)		
3	276 (2.4%)	76 (2.8%)	0.002	1.09
Feel disturbed by rail traffic				
noise	N = 11139	N = 2662		
0	10046 (90.2%)	2390 (89.8%)		
1	920 (8.3%)	232 (8.7%)		
2	135 (1.2%)	32 (1.2%)		
3	38 (0.3%)	8 (0.3%)	0.705	1.02
Feel disturbed by air traffic				
noise	N = 11211	N = 2690		
0	7629 (68.0%)	1727 (64.2%)		
1	2858 (25.5%)	734 (27.3%)		
2	556 (5.0%)	178 (6.6%)		
3	168 (1.5%)	51 (1.9%)	< 0.001	1.15
Feel disturbed by port noise	N = 11089	N = 2662		
0	10701 (96.5%)	2551 (95.8%)		
1	345 (3.1%)	103 (3.9%)		
2	39 (0.4%)	7 (0.3%)		
3	4 (0.0%)	1 (0.0%)	0.215	1.12
Feel disturbed by human noise -	N = 10995	N = 2627		
outdoors	7445 (67.7%)	1704 (64.9%)		
0	3118 (28.4%)	790 (30.1%)	< 0.001	1.13

1				
2	354 (3.2%)	101 (3.8%)		
3	78 (0.7%)	32 (1.2%)		
Feel disturbed by human noise - indoors	N = 11207	N = 2684		
0	8132 (72.6%)	1861 (69.3%)		
1	2699 (24.1%)	703 (26.2%)		
2	312 (2.8%)	90 (3.4%)		
3	64 (0.6%)	30 (1.1%)	< 0.001	1.16
Feel disturbed by animal noise	N = 11172	N = 2673		
0	10160 (90.9%)	2389 (89.4%)		
1	931 (8.3%)	257 (9.6%)		
2	67 (0.6%)	22 (0.8%)		
3	14 (0.1%)	5 (0.2%)	0.008	1.17
Feel disturbed by miscellaneous noise	N = 11070	N = 2628		
0	8268 (74.7%)	1819 (69.2%)		
1	2533 (22.9%)	689 (26.2%)		
2	206 (1.9%)	91 (3.5%)		
3	63 (0.6%)	29 (1.1%)	< 0.001	1.29

Table 20: Statistical analysis for noise at work using unadjusted logistic regression. Answers are given on a scale from 1 (Always) to 5 (Never).

Variable	No Tinnitus	Tinnitus	p-value	Odds Ratio
How often changing acustics at work?	N = 375	N = 228		
1	39 (10.4%)	26 (11.4%)		
2	138 (36.8%)	72 (31.6%)		
3	87 (23.2%)	68 (29.8%)		
4	88 (23.5%)	51 (22.4%)		
5	23 (6.1%)	11 (4.8%)	0.956	1.0
How often client/employee conversations?	N = 375	N = 227		
1	63 (16.8%)	35 (15.4%)		
2	196 (52.3%)	112 (49.3%)		
3	61 (16.3%)	42 (18.5%)		
4	27 (7.2%)	19 (8.4%)		
5	28 (7.5%)	19 (8.4%)	0.344	1.08
How often professional	N = 372	N = 226		
development opportunities?	6 (1.6%)	2 (0.9%)		
1	58 (15.6%)	29 (12.8%)		
2	135 (36.3%)	96 (42.5%)		
3	125 (33.6%)	72 (31.9%)		
4	48 (12.9%)	27 (11.9%)	0.943	1.01

5				
How often specific hygiene requirements?	N = 371	N = 227		
1	46 (12.4%)	24 (10.6%)		
2	56 (15.1%)	24 (10.6%)		
3	42 (11.3%)	21 (9.3%)		
4	88 (23.7%)	51 (22.5%)		
5	139 (37.5%)	107 (47.1%)	0.028	1.14
How often phone calls for work?	N = 377	N = 229		
1	55 (14.6%)	34 (14.8%)		
2	210 (55.7%)	130 (56.8%)		
3	61 (16.2%)	42 (18.3%)		
4	38 (10.1%)	16 (7.0%)		
5	13 (3.4%)	7 (3.1%)	0.488	0.94
How often have to understand speech in noisy environments?	N = 375	N = 225		
1	35 (9.3%)	29 (12.9%)		
2	130 (34.7%)	81 (36.0%)		
3	118 (31.5%)	71 (31.6%)		
4	70 (18.7%)	36 (16.0%)		
5	22 (5.9%)	8 (3.6%)	0.072	0.86
How often meetings with multiple colleagues?	N = 377	N = 228		
1	24 (6.4%)	10 (4.4%)		
2	168 (44.6%)	122 (53.5%)		
3	115 (30.5%)	64 (28.1%)		
4	50 (13.3%)	27 (11.8%)		
5	20 (5.3%)	5 (2.2%)	0.103	0.86
How often need to pay attention to warning signals/machine noise?	N = 362	N = 225		
1	20 (5.5%)	9 (4.0%)		
2	25 (6.9%)	18 (8.0%)		
3	47 (13.0%)	22 (9.8%)		
4	99 (27.3%)	55 (24.4%)		
5	171 (47.2%)	121 (53.8%)	0.219	1.1
How often NOT need to talk to others at work?	N = 370	N = 225		
1	0 (0.0%)	2 (0.9%)		
2	52 (14.1%)	40 (17.8%)		
3	84 (22.7%)	50 (22.2%)		
4	163 (44.1%)	97 (43.1%)		
5	71 (19.2%)	36 (16.0%)	0.115	0.87
How often other	N = 221	N = 140		
communication/hearing	46 (20.8%)	28 (20.0%)	0.257	0.91

requirements at work?		
1		
2	90 (40.7%)	63 (45.0%)
3	35 (15.8%)	26 (18.6%)
4	9 (4.1%)	7 (5.0%)
5	41 (18.6%)	16 (11.4%)
